# KDM6A promotes hepatocellular carcinoma progression and dictates lenvatinib efficacy by upregulating FGFR4 expression

**DOI:** 10.1002/ctm2.1452

**Published:** 2023-10-17

**Authors:** Wenyun Guo, Songling Li, Yifei Qian, Linfeng Li, Fan Wang, Yu Tong, Qianyu Li, Zijun Zhu, Wei‐Qiang Gao, Yanfeng Liu

**Affiliations:** ^1^ State Key Laboratory of Systems Medicine for Cancer Department of Liver Surgery Renji‐Med‐X Clinical Stem Cell Research Center, RenJi Hospital, School of Medicine, Shanghai Jiao Tong University Shanghai P. R. China; ^2^ School of Biomedical Engineering & Med‐X Research Institute Shanghai Jiao Tong University Shanghai P. R. China; ^3^ Shanghai Engineering Research Center of Transplantation and Immonology Shanghai Institute of Transplantation Shanghai P. R. China

**Keywords:** FGFR4, hepatocellular carcinoma, KDM6A, lenvatinib, lipid metabolism

## Abstract

**Background:**

Hepatocellular carcinoma (HCC) is one of the major causes of death from cancer and has a very poor prognosis with few effective therapeutic options. Despite the approval of lenvatinib for the treatment of patients suffering from advanced HCC, only a small number of patients can benefit from this targeted therapy.

**Methods:**

Diethylnitrosamine (DEN)‐CCL4 mouse liver tumour and the xenograft tumour models were used to evaluate the function of KDM6A in HCC progression. The xenograft tumour model and HCC cell lines were used to evaluate the role of KDM6A in HCC drug sensitivity to lenvatinib. RNA‐seq and ChIP assays were conducted for mechanical investigation.

**Results:**

We revealed that KDM6A exhibited a significant upregulation in HCC tissues and was associated with an unfavourable prognosis. We further demonstrated that KDM6A knockdown remarkably suppressed HCC cell proliferation and migration in vitro. Moreover, hepatic Kdm6a loss also inhibited liver tumourigenesis in a mouse liver tumour model. Mechanistically, KDM6A loss downregulated the FGFR4 expression to suppress the PI3K–AKT–mTOR signalling pathway, leading to a glucose and lipid metabolism re‐programming in HCC. KDM6A and FGFR4 levels were positively correlated in HCC specimens and mouse liver tumour tissues. Notably, KDM6A knockdown significantly inhibited the efficacy of lenvatinib therapy in HCC cells in vitro and in vivo.

**Conclusions:**

Our findings revealed that KDM6A promoted HCC progression by activating FGFR4 expression and may be an essential molecule for influencing the efficacy of lenvatinib in HCC therapy.

## Graphical abstract

1. KDM6A is upregulated in HCC, linked to a poor prognosis and affects HCC cell growth *in vitro and in vivo*.

2. KDM6A regulates the expression of FGFR4 to activate the PI3K–AKT–mTOR pathway, leading to a glucose and lipid metabolism re‐programming in HCC.

3. KDM6A may be an essential molecule to influence the efficacy of lenvatinib for HCC therapy.

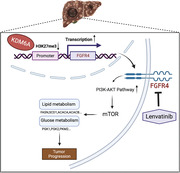

## 1 INTRODUCTION

Hepatocellular carcinoma (HCC), as the fourth leading cause of cancer‐related death globally,^1^ has a very poor prognosis with a 3‐year survival rate of about 12% and a median survival period of less than a year.^2^ Lenvatinib, a new targeted drug, has recently been approved as a first‐line treatment option for patients suffering from advanced‐stage HCC.^3^ However, only 24.1% of patients can benefit from the lenvatinib‐targeted therapy.^4^ Therefore, identifying effective biomarkers for predicting responsiveness in patients with HCC receiving lenvatinib therapy is desired.

Fibroblast growth factor receptors (FGFRs) comprising FGFR1,FGFR2,FGFR3 and FGFR4 have been reported to play important roles in the regulation of the main target genes of lenvatinib and control tumour metabolism in many cancer types including HCC.^5^ Particularly, FGFR4 is highly expressed in liver tissues and is an emerging target for HCC. Furthermore, its activation modulates multiple signalling cascades, including PI3K–AKT–mTOR signalling pathway and RAS–RAF–MAPK signalling pathway to promote HCC initiation and progression.^6^, ^7^ So far, most studies have mainly focused on the aberrant activation of FGF19–FGFR4 signalling in HCC,^8^, ^9^ but the regulation mechanism of FGFR4 expression in tumour progression is still largely unknown. Therefore, investigating the underlying molecular mechanisms of FGFR4 upstream regulation, is crucial in enhancing the lenvatinib efficiency and prolonging the survival rate of patients with advanced disease.

Lysine demethylase 6A (KDM6A) is a member of the KDM family of histone H3 lysine 27 demethylases, which plays an essential role in cell differentiation.^10^, ^11^ Moreover, KDM6A plays a vital role in the tumour progression of various cancer types such as colorectal cancer, breast cancer and bladder cancer and serves as a tumour suppressor or oncogene, depending on the cancer types.^12^, ^13^, ^14^, ^15^ However, whether KDM6A participates in the progression of HCC remains unclear.

In this study, we demonstrated that KDM6A expression was upregulated in HCC and was positively associated with a poor prognosis. Moreover, KDM6A loss inhibited HCC cell growth in vitro and in vivo. Importantly, we revealed that KDM6A knockdown significantly reduced the sensitivity of HCC cells to lenvatinib treatment by regulating FGFR4 expression, indicating that patients suffering from HCC with a low KDM6A expression may be more resistant to lenvatinib‐targeted therapy.

## 2 METHODS AND MATERIALS

### 2.1 Plasmids and reagents

The shKDM6A (short hairpin RNA) targeting the CDS region (human, same in mouse) and 3′UTR region (for rescue experiments) was cloned into the pLKO.1 vector. The shKDM6A and shFgfr4 sequences are presented in Supporting Information Table S1. Human KDM6A was cloned into the lentiviral vector H105 pLenti‐CMV‐mCherry–3FLAG–PGK–Puro to generate the corresponding expression plasmids.

All constructs were confirmed by sequencing. For lentivirus infection, psPAX2 (#12260, addgene) and pMD2.G (#12259, addgene) in a ratio of 2:1:1 (target plasmids: psPAX2: pMD2.G) were used. Lentivirus‐transduced cells expressing the puromycin resistance gene were subjected to selection with 5 μg/mL puromycin for 7 days.

### 2.2 Establishment of KDM6A conditional knockout mice

Genotypes in this study included KDM6A^flox/Y^ Alb‐Cre+ and KDM6A ^flox/Y^Alb‐Cre‐. Mice carrying conditional alleles of KDM6A^flox/Y^ were provided from Shanghai Model Organisms Center, Inc. All animal experiments were conducted in the compliance with the guidelines and regulations set forth by Shanghai Renji Hospital Animal Care and Use Committee. The primers used for genotyping and qPCR validation are shown in Supporting Information Table S1.

### 2.3 HCC mouse model

For diethylnitrosamine (DEN)‐CCL4 mouse model establishment, male mice (*Kdm6a flox/Y*) were administered a single injection (i.p.) of 25 mg/kg DEN at 14 days of age. Thereafter, mice were administered 100 μL CCl4 (25%) i.p. one time per week for up to 12 weeks,^16^ starting at 8 weeks of age. Mice were then sacrificed, and livers and sera were collected for subsequent analysis. Tumour burden = tumour area/liver area × 100%.

### 2.4 Mice treatment

Male C57 mice (aged 5−6 weeks, www.jh‐labanimal.site/) were used. We performed tumour graft experiment by injecting 1 × 10^6^ Hepa1–6 cells (SCR or cells infected with the shKdm6a‐1 virus). After 3 days, sub‐cutaneous tumours achieved a measurable size. Lenvatinib (HY‐10981; MCE) was dissolved in DMSO and then the solvent control and lenvatinib were diluted in corn oil and given intragastrically at a dosage of 10 mg/kg every 2 days to be used in drug sensitivity experiments. After 16 days, the mice were sacrificed using cervical dislocation. Then, we removed tumours for analysis. The tumour volume = length × width^2^/2.

### 2.5 Dual‐luciferase reporter assays

HET293T cells were plated in 24‐well plates at a concentration of 60%. A total of 300 ng of FGFR4 promoter reporter plasmid (P1–P5) or pGL3 vector and 25 ng of Renilla plasmid with 300 ng shKDM6As or vector plasmid were co‐transfected per well. After 48 h, we used a dual‐luciferase reporter assay kit (Vazyme, DL101) and quantified it by Junior LB 9509 (Berthold Technologies) to evaluate luciferase activities based on the manufacturer's instructions. For each well, the luciferase activity was adjusted to Renilla luciferase. The promoter plasmid sequences are seen in Supporting Information Table S1.

### 2.6 Chromatin immunoprecipitation‐qPCR (ChIP‐qPCR)

We performed the chromatin immunoprecipitation (ChIP) assay by the Enzymatic Chromatin IP Kit (Cell Signaling, #9003S) according to the manufacturer's instructions. Antibody H3k27me3 (ABclonal, A2363,1:100) was used here. We performed qPCR to analyse the eluted DNA fragments. The primers we used are listed in Supporting Information Table S1

### 2.7 Immunohistochemistry (IHC) and human HCC tissue microarray

Mice liver tissues were fixed in 4% paraformaldehyde at room temperature, then embedded in paraffin and sliced into 2‐μm thick sections. For antigen retrieval, we used sodium citrate buffer (pH 6.0 for Fgfr3, Fgfr4 and PCNA) and buffer (pH = 8.0 for Kdm6a, Scd1, p70‐S6k1, p‐Akt and Fasn). And then the slides were boiled in a steamer for 20 min, and cooled down at room temperature. Then tissues were permeabilised with 0.05% Triton ×‐100 for 15 min at room temperature. Then tissues were blocked with 5% donkey serum in PBS for 1 h. Then the sections were incubated with primary antibodies overnight at 4˚C. Following the washing step with PBS, the sections were incubated with secondary antibodies for a duration of 1 h the next day. Staining was visualized using 3,3′‐diaminobenzidine (DAB) and counterstained with haematoxylin.

For human HCC tissues immunohistochemistry (IHC), a total of 30 paired primary HCC tissues and adjacent normal tissues were obtained from patients (age 24−71 years, 20 male and 10 female patients, diameter of tissue: 1.0 mm, from Alenabio Company (www.alenabio.com/).

A total of 90 HCC patient tissues (age 33−83 years, 72 male and 18 female patients, diameter of tissue: 1.5 mm) were randomly collected from Shanghai Renji Hospital between 2014 and 2018, and tissue microarrays were prepared by the Shanghai Zuocheng Biocompany. Every participant signed informed consent before participating in the experiments and analyses. The Research Ethics Committee of Shanghai Renji Hospital granted approval for all the manipulations performed during the study.

The staining extent score was assigned on a scale of 0−8, according to the percentage of positive staining cells (0−100%) and the staining intensity, where 0 indicated negative and 8 indicated very strong staining. Further, we multiplied the staining extent score with the staining intensity score of each specimen to calculate IHC scores. The rectangles within the figure signified a magnified image (40×).

### 2.8 Bioinformatics analysis

We collected the expression data of KDM6A, FGFR1, FGFR2, FGFR3 and FGFR4 and clinical information from TCGA_LIHC dataset (https://portal.gdc.cancer.gov/projects/TCGA‐LIHC) and GSE22058, GSE76311, GSE14520, GSE148355, GSE25097, GSE186191. The IC50 of Lenvatinib and KDM6A expression in HCC cell lines were collected from Liver Cancer Model Repository (LIMORE)^17^ (http://www.picb.ac.cn/limore/). IHC staining intensity collected from PronteinAtlas (https://www.proteinatlas.org/). CHCC‐ hepatitis B virus (HBV) dataset^18^ contained the mRNA and protein information of HBV‐related paired tumour and adjacent HCC tissues from 159 patients. We obtain the genomic data from the National Omics Data Encyclopedia (NODE).

Drug sensitivity was analysed using the ‘OncoPredict’ R package (R 4.2.1). We used TCGA_LIHC datasets as test Expr Data and gained data including drug sensitivity scores. Then we analysed the correlation between KDM6A expression and drug prediction scores. Detailed information is in Supporting Information Table S2.

### 2.9 Statistical analysis

We performed statistical analyses using Prism 8.0 (GraphPad Software). Mice tumour burdens were calculated by the Fiji ImageJ software. A two‐tailed unpaired Student *t* test was applied for comparisons between two groups. The significance of multiple group comparisons was analysed by one‐way ANOVA. The Kaplan–Meier plot was used for patients. The log‐rank test was used to examine the difference in survival rates. *p < .05; **p < .01; ***p < .001 and ****p < .0001 were used as indicators of statistical significance. Additional material and methods are presented in Supporting Information.

## 3 RESULTS

### 3.1 KDM6A expression is unregulated in HCC and is associated with a worse prognosis

**FIGURE 1:**
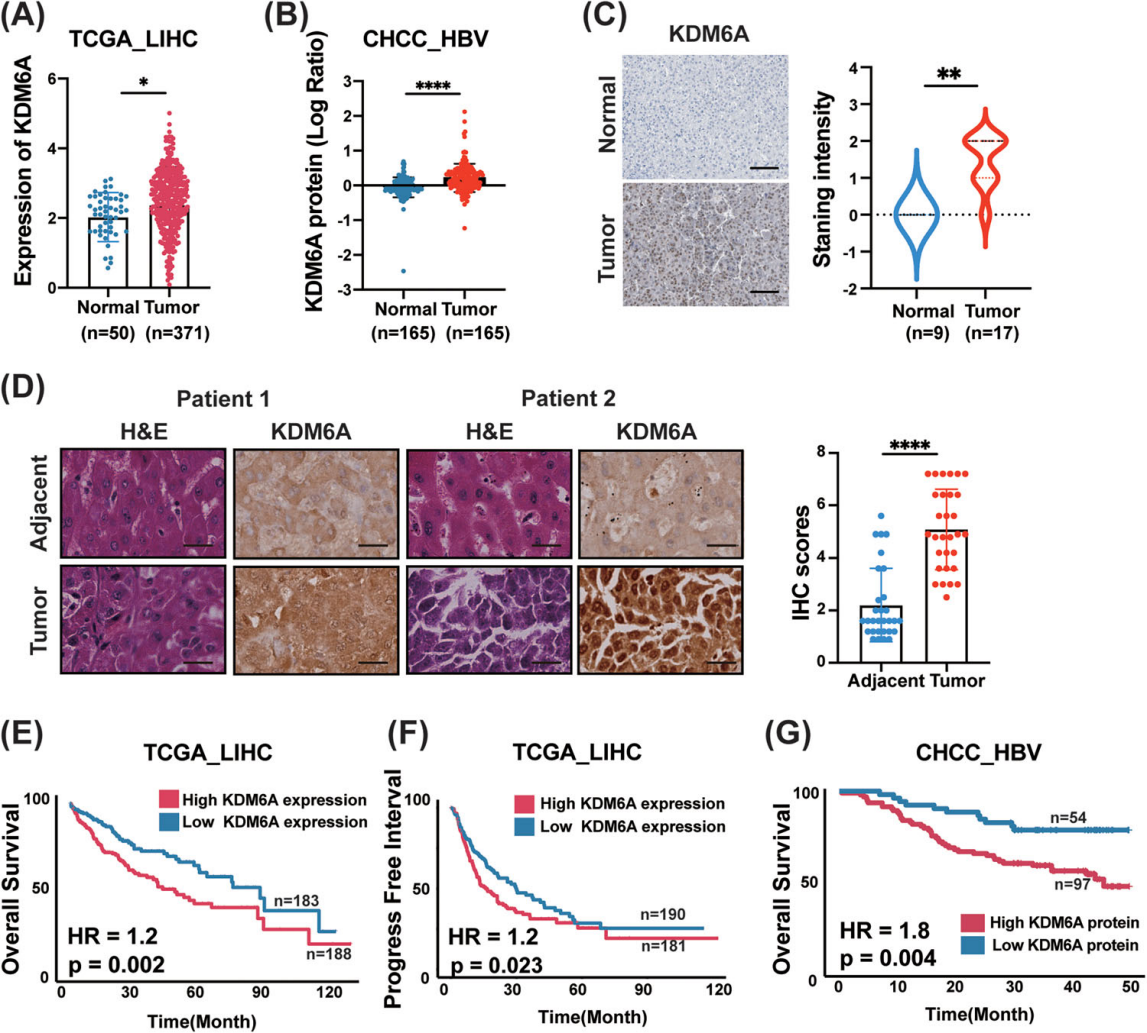
KDM6A is abundantly expressed in tumour tissues and is associated with a poor prognosis. **(A)** Comparison of the KDM6A mRNA level between HCC (Tumour) and normal tissues (Normal) in TCGA_LIHC dataset. TCGA, The cancer genome atlas; LIHC, liver hepatocellular carcinoma. **(B)** Comparison of the KDM6A protein level between HCC (Tumour) and normal tissues (Normal) in CHCC_HBV dataset. CHCC_HBV, HBV‐related hepatocellular carcinoma dataset, see Section 2. **(C)** Staining intensity of the KDM6A protein level between HCC (Tumour) and normal tissues (Normal) in PronteinAtlas (https://www.proteinatlas.org/). Scale bar,100 μm. **(D)** Staining intensity of the KDM6A protein level between HCC (Tumour) and normal tissues (Normal) in HCC tissue microarray (30 HCC tissues and 30 adjacent normal tissue). Representative images were shown (Patient 1 and Patient 2) and quantification results. The IHC scores were assigned according to the percentage of positive staining cells (0−100%) and the staining intensity (0–8). Scale bar, 25 μm. **(E‐F)** Kaplan–Meier analysis of overall survival (OS) probability **(E)** and progression‐free interval probability **(F)** of KDM6A levels in TCGA_LIHC patients. The statistical significance was assessed using two‐sided log‐rank test. TCGA, The cancer genome atlas; LIHC, liver hepatocellular carcinoma. **(G)** Kaplan–Meier analysis of OS probability of KDM6A protein levels in CHCC_HBV patients. The statistical significance was assessed using two‐sided log‐rank test. CHCC_HBV, HBV‐related hepatocellular carcinoma dataset, see Section 2. Data represent mean ± SD. *p* Values were calculated using a student *t* test unless otherwise stated. *p < .05; **p < .01; ***p < .001; ****p < .0001.

To evaluate the possible roles of KDM6A in HCC progression, we first analysed the expression of KDM6A in the HCC tissues from the TCGA_LIHC cohort and found that KDM6A was upregulated in liver tumour tissues compared to the normal liver tissues (Figure 1A). Similar results were observed in several other HCC cohorts from the Gene Expression Omnibus (GEO) databases (GSE22058, GSE76311, GSE14520 and GSE25097) (Supporting Information Figure S1A, B). Furthermore, KDM6A expression was positively correlated with higher tumour grades in the HCC cohort from the GEO database (GSE148355) ,^19^ suggesting that KDM6A is associated with clinically malignant potential (Supporting Information Figure S1C). Furthermore, we found that the protein level of KDM6A was upregulated in HCC tissues in an HBV‐related HCC cohort (CHCC_HBV)^18^ (Figure 1B). Consistently, the IHC staining intensity of KDM6A (Proteinatlas, https://www.proteinatlas.org/) was also higher in the tumour tissues compared with the normal liver tissues (Figure 1C). To further validate these results, we performed IHC staining on HCC tissues to evaluate the protein expression of KDM6A in human HCC and normal tissue samples. Notably, these in‐house HCC samples also displayed a stronger staining intensity than the corresponding normal tissues (Figure 1D). Also, we examined Kdm6a protein levels in normal liver tissues and HCC tissues from the DEN mouse model, and we found that liver tumour tissues displayed higher KDM6A protein levels compared to normal liver tissues (Supporting Information Figure S1D).

Next, to investigate the relationship between KDM6A expression and clinical outcomes, we first divided all HCC samples in the TCGA_LIHC cohort into a high KDM6A expression group and a low KDM6A expression group based on the median mRNA level of KDM6A. Then, we performed Kaplan–Meier analyses. We found that the high KDM6A expression group displayed a shorter overall survival (OS) and progression‐free interval time compared to patients with low KDM6A expression (Figure 1E, F). Consistently, we also found that high KDM6A protein levels could result in a shortened OS based on the analysis of the CHCC_HBV dataset (Figure 1G). A similar result was also found in the 90 HCC patients’ cohort from Renji hospital (Supporting Information Figure S1E).

Given that previous studies have reported that KDM6A is a gender‐specific molecular and can escape chromosome × inactivation.^20^ Therefore, we examined whether gender had any effect on the KDM6A expression in males and females suffering with HCC. As expected, higher expression of KDM6A was observed in females in the TCGA_LIHC and CHCC_HBV datasets (Supporting Information Figure S1F). Additionally, Kaplan–Meier survival curves grouped by sex demonstrated a relatively worse survival in females (high KDM6A expression) than in males (low KDM6A expression) according to the TCGA_LIHC and CHCC_HBV datasets (Supporting Information Figure S1G). In agreement with the above‐described findings, similar results were obtained based on the analysis using the TCGA_LIHC database in separated genders (Supporting Information Figure S1H–M).

### 3.2 KDM6A affects HCC cell growth and migration

**FIGURE 2:**
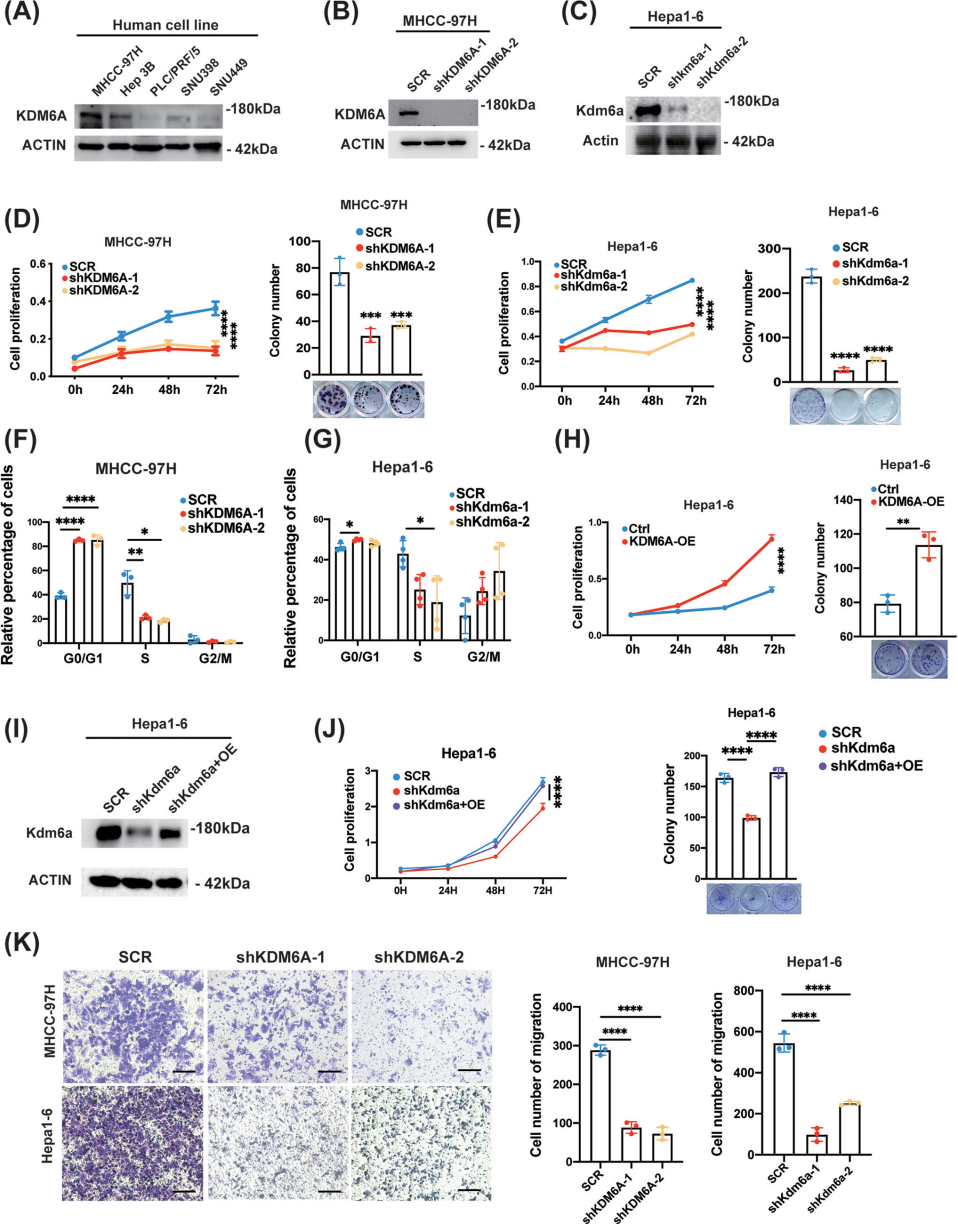
KDM6A knockdown reduces HCC cell proliferation and migration. **(A)** Western blot analysis of the protein levels of KDM6A in multiple HCC cell lines (MHCC‐97H, Hep 3B, PLC/PRF/5, SNU398 and SNU449). HCC, hepatocellular carcinoma. **(B‐C)** Western blot analysis of the protein levels of KDM6A after stable knockdown of KDM6A by shRNAs in MHCC‐97H and Hepa1–6 cell lines. **(D‐E)** CCK8 assays (n = 6) and colony formation assays (n = 3) of MHCC‐97H **(D)** and Hepa1–6 **(E)** cells after stable knockdown of KDM6A (n = 6). CCK8, Cell Counting Kit‐8. **(F‐G)** Cell cycle analysis of MHCC‐97H (n = 3) **(F)** and Hepa1–6 (n = 4) **(G)** cells after KDM6A stable knockdown. **(H)** CCK8 assay (n = 6) and colony formation assay (n = 3) of Hepa1–6 cells after stable overexpression of KDM6A. **(I)** Validation of KDM6A expression by Western blot in Kdm6a stable knockdown (target 3′UTR region) Hepa1–6 cells after forced Kdm6a overexpression. **(J)** CCK8 assay (n = 6) and colony formation assay (n = 3) in Kdm6a stable knockdown (target 3′UTR region) Hepa1–6 cells after forced Kdm6a overexpression. **(K)** Transwell assays of MHCC‐97H and Hepa1–6 cells after stable knockdown of KDM6A by shRNAs. Representative images (left) and quantification results (n = 3) were shown. Scale bar, 25 μm. Data represent mean ± SD. *p* Values were calculated using a student *t* test. The significance of multiple group comparisons was analysed by one‐way ANOVA. *p < .05; **p < .01; ***p < .001; ****p < .0001.

To verify the functions of KDM6A in HCC cell growth and migration, we conducted knockdown experiments. To achieve this goal, we first measured the protein levels of KDM6A in multiple human HCC cell lines (including MHCC‐97H, Hep 3B, PLC/PRF/5, SNU398 and SNU449) using Western blotting (Figure 2A). We then selected human MHCC‐97H and Hep 3B cells, which exhibited higher KDM6A expression levels compared with other HCC cell lines and mouse Hepa1–6 cells, to construct stable KDM6A knock‐down cell lines for further functional research. As shown in Supporting Information Figure S2A, B and C, KDM6A levels were stably downregulated in human MHCC‐97H and Hep 3B cells as well as mouse Hepa1–6 cells by the two shRNAs. Using the CCK‐8 assays, we found that KDM6A deficiency in human MHCC‐97H and Hep 3B cells and mouse Hepa1–6 cells dramatically inhibited HCC cell proliferation. In addition, the colony formation assays revealed that KDM6A knockdown also reduced the cell colonies number in human MHCC‐97H and Hep 3B cells and mouse Hepa1–6 cells (Figure 2D, E and Supporting Information Figure S2C).

As cell‐cycle phase transition is essential for cancer cell growth, we next investigated the changes in the cell cycle phases influenced by KDM6A deletion. We first performed gene set enrichment assay (GSEA) between the high KDM6A expression and low KDM6A expression group in the TCGA_LIHC dataset (Supporting Information Figure S2D). Then, we examined the cell cycle distribution in HCC cells after KDM6A knockdown. As expected, the downregulation of KDM6A prevented the cell cycle progression from the G0/G1 to S phase in human MHCC‐97H and mouse Hepa1–6 cells (Figure 2F,G).

To determine if KDM6A still keeps its functions in KDM6A‐lowly expressing cells, we performed knockdown experiments in PRF/PLC/5 cells, which expressed the lowest levels of KDM6A among all of five HCC cells examined (Figure 2A). First, qPCR and western blotting confirmed a further reduction of KDM6A mRNA and protein levels (Supporting Information Figure S2E, F). Then we observed that knockdown of KDM6A still inhibited the cell proliferation ability in PRF/PLC/5 cells (Supporting Information Figure S2G). We think that the reason why knocking down KDM6A in the lowly expressing PLC/PRF/5 cells still showed the tumour growth‐inhibiting effects was presumably due to the residual expression of KDM6A in PLC/PRF/5, which remains to function to a large extent to promote PLC/PRF/5 cells proliferation ability.

To perform a gain‐of‐function experiment, mouse Hepa1–6 cells were transfected with an empty or a KDM6A overexpression vector to construct a stable Kdm6a‐overexpressing cell line. The transfection efficiency of Kdm6a overexpression was confirmed using qPCR and western blotting (Supporting Information Figure S2H,I). As expected, Kdm6a overexpression promoted Hepa1–6 cell proliferation according to both the CCK8 assays and colony formation assays (Figure 2H). Moreover, to rule out a possibility of an off‐target effect by the shRNA, we performed the rescue experiments to validate the specific function of KDM6A. We found that forced KDM6A expression could eliminate the cellular effect of KDM6A knockdown (targeting the 3′UTR region) in human PLC/PRF/5 and mouse Hepa1–6 stable overexpression cell lines (Figure 2I, J and Supporting Information Figure S2J).

To provide additional evidence to support the HCC growth and progression‐promoting effects of KDM6A, we performed GSEA of TCGA_LIHC dataset and revealed that ‘Liver_cancer_up’ and ‘Metastasis_up’ pathways were significantly enriched in the high KDM6A expression group (Supporting Information Figure S2K). To validate the metastasis‐promoting effects of KDM6A, we performed a transwell assay using MHCC‐97H and Hepa1–6 cells. As shown in Figure 2K, KDM6A knockdown in MHCC‐97H and Hepa1–6 cells indeed reduced the number of migrating cells. Collectively, these results demonstrate that KDM6A promotes HCC cell growth and metastasis.

### 3.3 KDM6A loss suppresses liver tumourigenesis in a mouse HCC model

**FIGURE 3:**
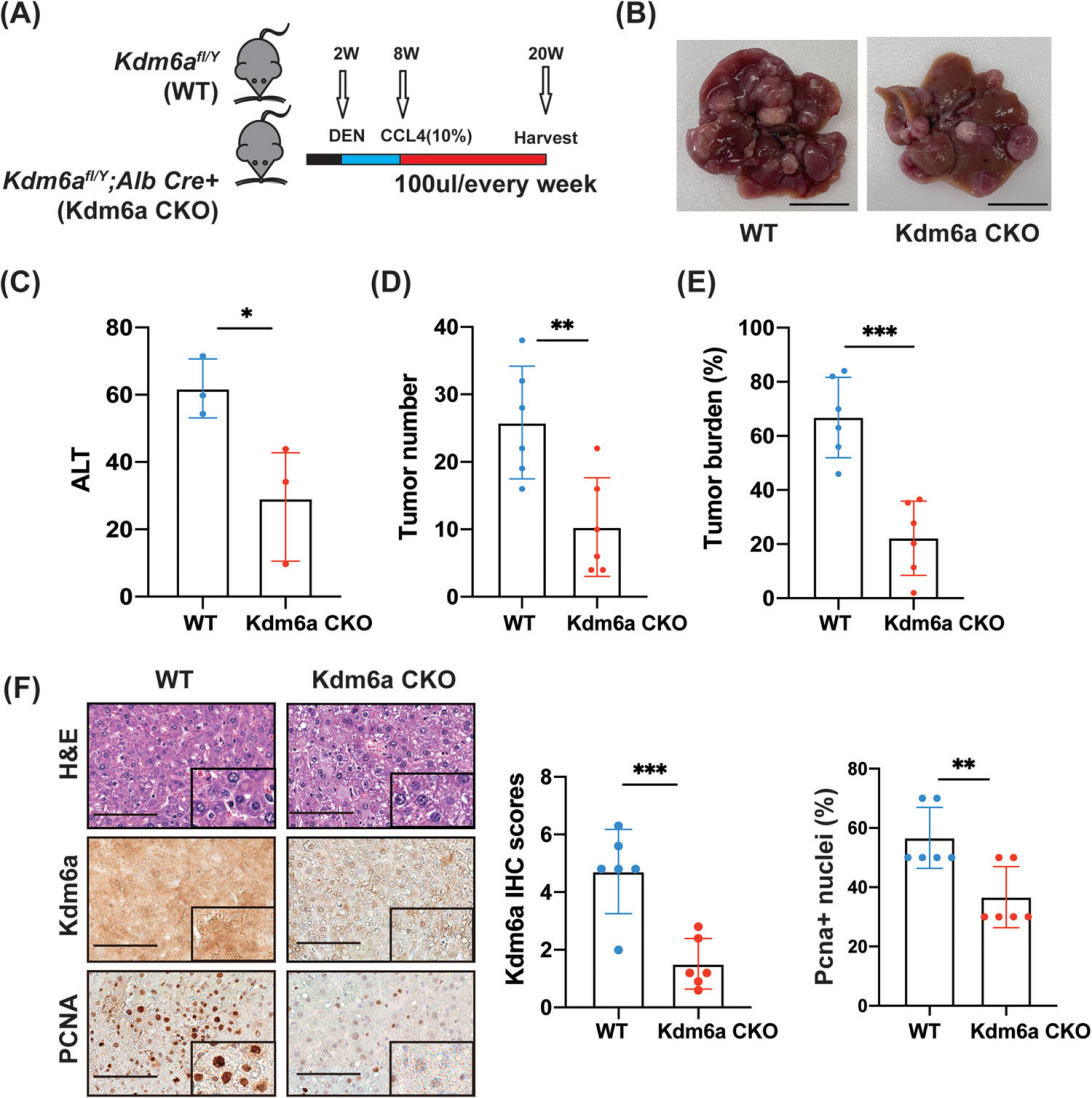
KDM6A promotes HCC tumourigenesis potential. **(A)** Schematic plot about establishment of mouse liver cancer model. **(B)** Representative images of HCC (n = 6 per group, WT versus Kdm6a CKO) from the mouse liver cancer models 12 weeks after HCC mouse model established (see Section 2). Scale bar,1 cm. **(C)** Serum ALT per mouse was measured (n = 3 per group, WT versus Kdm6a CKO). ALT, alanine transaminase. **(D)** Tumour number per mouse were measured (n = 6 mice per group, WT versus Kdm6a CKO). **(E)** Tumour burden per mouse liver were measured (n = 6 mice per group, WT versus Kdm6a CKO). Tumour burden = average (tumour area/liver area × 100%). **(F)** Representative images of IHC and H&E of Kdm6a, PCNA staining and the relative IHC scores (n = 6, WT versus Kdm6a CKO) of PCNA in mouse tumour tissues shown in **(B)**. The IHC scores were assigned according to the percentage of positive staining cells (0−100%) and the staining intensity (0–8). Scale bar, 100 μm. The rectangles within the figure panels signified a magnified image (40×). IHC, immunohistochemistry; H&E, haematoxylin–eosin staining. Data represent mean ± SD. *p* Values were calculated using a student *t* test unless otherwise stated. *p < .05; ** *p* < .01; ***p < .001.

To further explore the function of KDM6A in vivo, we constructed an *Alb‐cre; Kdm6a ^flox/Y^* (Kdm6a CKO) mouse model of tumourigenesis (Figure 3A, Supporting Information Figure S3A,B). Knockout efficiency was validated using a real‐time PCR assay (Supporting Information Figure S3C,D). Our results revealed that Kdm6a liver‐specific knockout (Kdm6a CKO) mice displayed a lower serum alanine transaminase (ALT) activity, fewer tumours and a lower tumour burden than wild‐type (WT) mice (Figure 3B–E). In addition, the body and liver weights were not significantly different between Kdm6a CKO and WT mice (Supporting Information Figure S3E). Furthermore, we also observed a reduction in the proliferative activity marker PCNA in the Kdm6a‐deficient livers according to IHC staining of mouse liver tissues (Figure 3F). In conclusion, these findings demonstrate that Kdm6a facilitates the hepatocarcinogenesis in mice.

### 3.4 KDM6A activates FGFR signalling by upregulating FGFR4 expression

**FIGURE 4:**
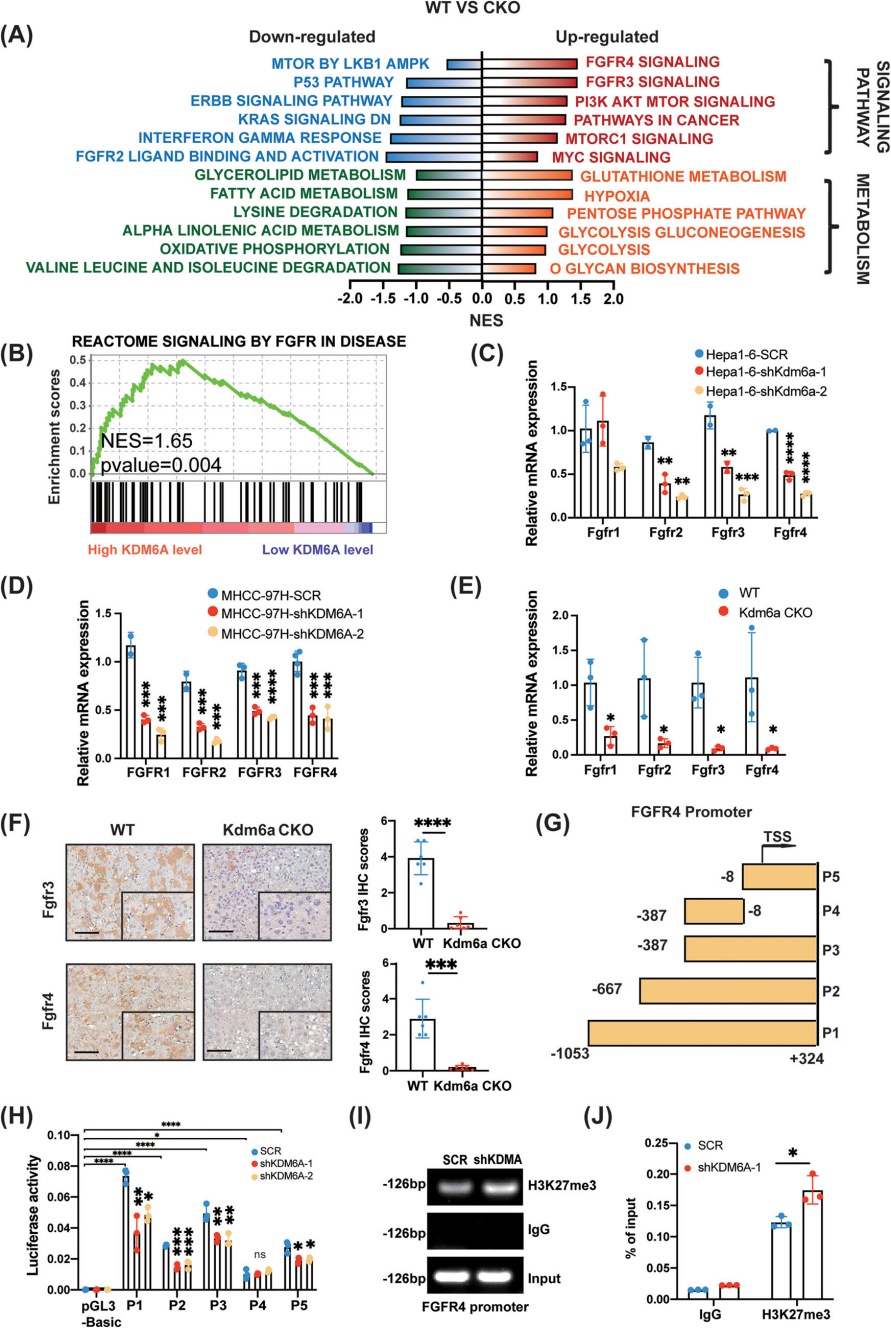
KDM6A regulates FGFR4 transcription to alter HCC metabolism. **(A)** The results of GSEA analysis in RNA‐sequencing (WT versus Kdm6a CKO, n = 5/5) in HCC tumour tissues. GSEA, Gene Set Enrichment Analysis. **(B)** The detailed results of enriched pathways related to Signaling By Fgfr in disease in TCGA_LIHC (KDM6A high level versus low, n = 185/184). TCGA, The Cancer Genome Atlas; LIHC, liver hepatocellular carcinoma. **(C‐E)** Relative mRNA level of FGFRs genes by qPCR in Hepa1–6 **(C),** MHCC‐97H **(D)** and mouse HCC tissues (WT versus Kdm6a CKO) **(E)** after KDM6A knockdown (n = 3). **(F)** Representative images of IHC and H&E of Fgfr3 and Fgfr4 staining and the relative IHC scores (n = 6) in mouse tumour tissues. The IHC scores were assigned according to the percentage of positive staining cells (0−100%) and the staining intensity (0–8). Scale bar, 100 μm. The rectangles within the figure panels signified a magnified image (40×). IHC, Immunohistochemistry; H&E, haematoxylin–eosin staining. **(G‐H)** Luciferase activities of corresponding fragments pGL3‐basic and (P1–P5) of FGFR4 promoter were examined by luciferase reporter assay in HET293T cells (n = 3, others see Section 2). **(I‐J)** ChIP assays above to examine the enrichment in FGFR4 promoter among indicated shKDM6A‐1 cells using antibodies of IgG and H3K27me3 (n = 3). Data represent mean ± SD. *p* values were calculated using a student *t* test. The significance of multiple group comparisons was analysed by one‐way ANOVA. ns, no significance; * *p* < .05; **p < .01; ***p < .001; ****p < .0001.

To elucidate the potential mechanism by which KDM6A mediates liver tumourigenesis, we performed RNA sequencing of liver tumour tissues from WT and Kdm6a CKO mice. GSEA showed that FGFR‐ and metabolism‐related pathways were significantly enriched (Figure 4A).

FGFRs, a subfamily of receptor tyrosine kinases (RTKs), are master regulators of a wide range of cellular processes including cell apoptosis, cell proliferation and angiogenesis.^21^ Therefore, we evaluated whether KDM6A could regulate the expression of FGFRs to activate the FGFR signalling, leading to liver tumourigenesis. We also performed GSEA of TCGA_LIHC dataset and revealed that the FGFR signalling elements were enriched in the high KDM6A expression group compared to the low KDM6A expression group (Figure 4B). In addition, the WT group showed higher FGFR signalling activity than the Kdm6a CKO group (Supporting Information Figure S4A).

Considering that the FGFR family consists of FGFR1, FGFR2, FGFR3, and FGFR4,^22^ we further examined whether KDM6A could upregulate FGFR1−4 expression. As expected, real‐time PCR results revealed that KDM6A knockdown significantly reduced the expression of FGFR2, FGFR3 and FGFR4 in human MHCC‐97H and mouse Hepa1–6 cells (Figure 4C,D). Similar results were obtained for liver tumour tissues in Kdm6a CKO mice (Figure 4E). Meanwhile, Kdm6a overexpression upregulated the FGFR1‐4 levels in Hepa1–6 cells (Supporting Information Figure S4B). Moreover, our RNA sequencing revealed a strong positive correlation between Kdm6a and Fgfr1, Fgfr3 and Fgfr4 expression with the exception of FGFR2, which displayed a negative correlation (Supporting Information Figure S4C). In addition, we found that FGFR2, FGFR3 and FGFR4 expression was increased in the tumour tissues compared to that in normal tissues in the TCGA_LIHC datasets (Supporting Information Figure S4D). Further, IHC assay was used to evaluate the protein levels of Fgfr3 and Fgfr4 in tumour sections derived from WT and Kdm6a CKO mouse HCC tissues. As expected, the IHC scores of Fgfr3 and Fgfr4 were decreased in Kdm6a‐deficient tumour tissues (Figure 4F), confirming that FGFR3 and FGFR4 expression was positively associated with KDM6A expression.

Thinking that FGFR4 served as a more important indicator of a worse prognosis in HCC of the TCGA‐LIHC database (Supporting Information Figure S4E), we further investigated the molecular mechanism of KDM6A‐mediated FGFR4 upregulation. According to the distribution of transcription initiation sites (TSS), acetylation sites and cis‐acting elements on the gene, we constructed different FGFR4 promoter vectors (P1: −1053/+324; P2: −667/+324; P3: −387/+324; P4: −387/−8 and P5: −8/+324) to elucidate the relationship between KDM6A and FGFR4 expression and transfected them into HEK293T cells. Our results revealed that KDM6A knockdown could reduce the activity of FGFR4 promoter fragments of P1–P3, P5 (P1: 1377 bp; P2: 991 bp; P3: 711 bp and P5: 331 bp), but not P4 (P4: 379 bp), as determined using a dual‐luciferase reporter experiment (Figure 4G,H), suggesting that the region (−8 to +324) in the FGFR4 promoter was mainly regulated by KDM6A. Further, we performed ChIP assays and qPCR to analyse eluted DNA fragments. As expected, we found the immunoprecipitation of the activated region of the FGFR4 promoter with anti‐H3K27me3 antibodies and that KDM6A knockdown led to an increased association between the FGFR4 gene promoter and H3K27me3 (Figure 4I,J). These findings indicated that KDM6A is critical for FGFR4 transcription.

### 3.5 KDM6A is positively associated with FGFR4 expression in HCC tissues

**FIGURE 5:**
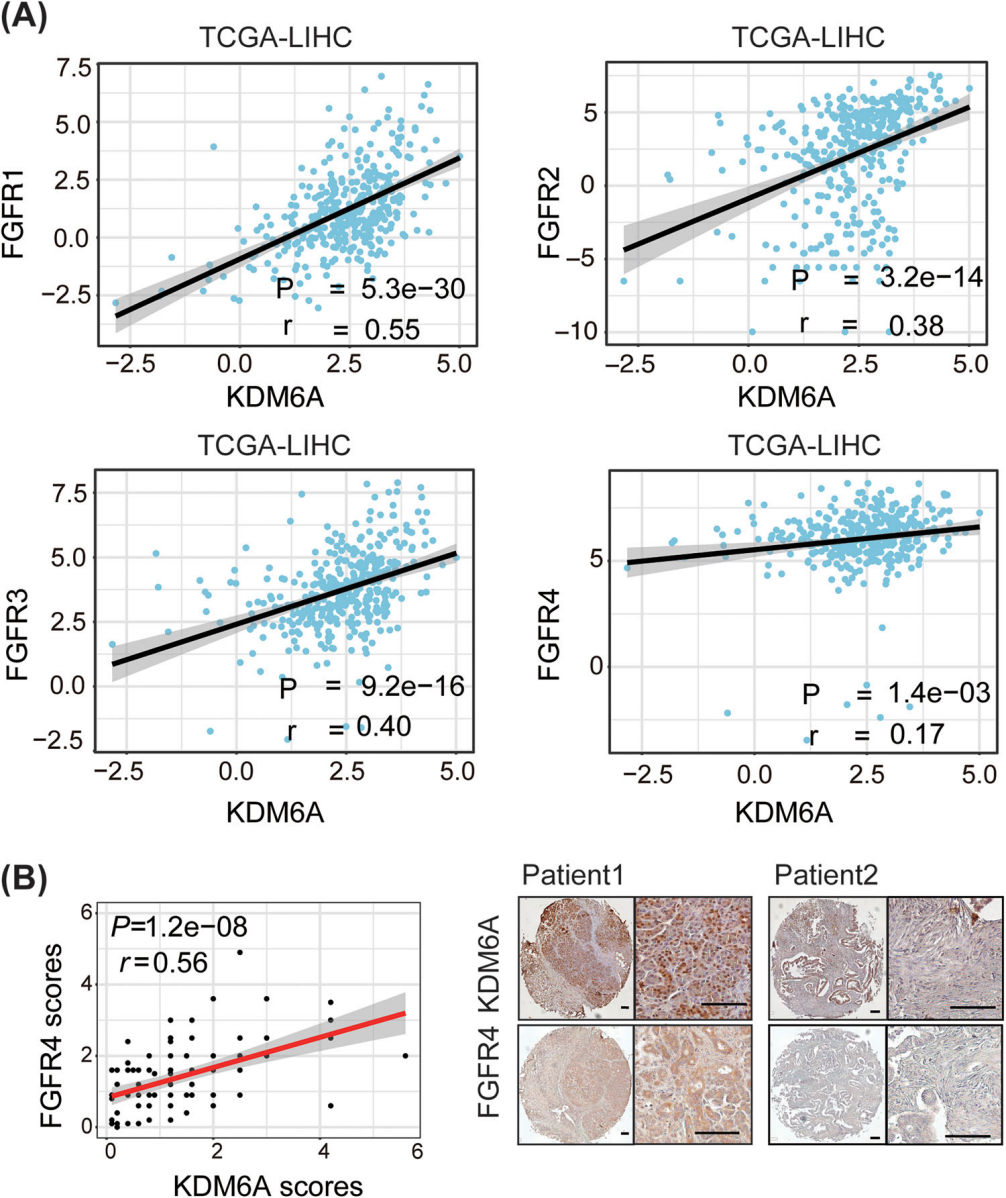
KDM6A expression is positively correlated to FGFR4 expression in clinical samples. **(A)** Correlation between KDM6A and FGFRs mRNA level in TCGA_LIHC by Spearman's correlation coefficient analysis. TCGA, The Cancer Genome Atlas; LIHC, liver hepatocellular carcinoma. **(B)** Representative images (right) of IHC of KDM6A and FGFR4 staining in human HCC microarray and the correlation (left) between KDM6A and FGFR4 IHC scores by Spearman's correlation coefficient analysis (n = 90). The IHC scores were assigned according to the percentage of positive staining cells (0−100%) and the staining intensity (0–8). IHC, Immunohistochemistry. Scale bar, 100 μm.

To clarify whether KDM6A regulates FGFRs in clinical samples, we analysed the relationship between KDM6A and FGFRs expression in the TCGA‐LIHC and CHCC_HBV cohorts. Spearman's correlation analysis revealed a positive correlation between KDM6A and FGFR expression (Figure 5A and Supporting Information Figure S5). To further study the clinical relevance of KDM6A and FGFR4 in HCC, we evaluated the protein expression of KDM6A and FGFR4 by IHC scores. Consistently, IHC staining result showed a positive correlation between FGFR4 and KDM6A protein levels in HCC tissues (Figure 5B), indicating that KDM6A upregulates FGFR4 expression in HCC tissues.

### 3.6 KDM6A regulates lipid and glycose metabolism programming in HCC cells

**FIGURE 6:**
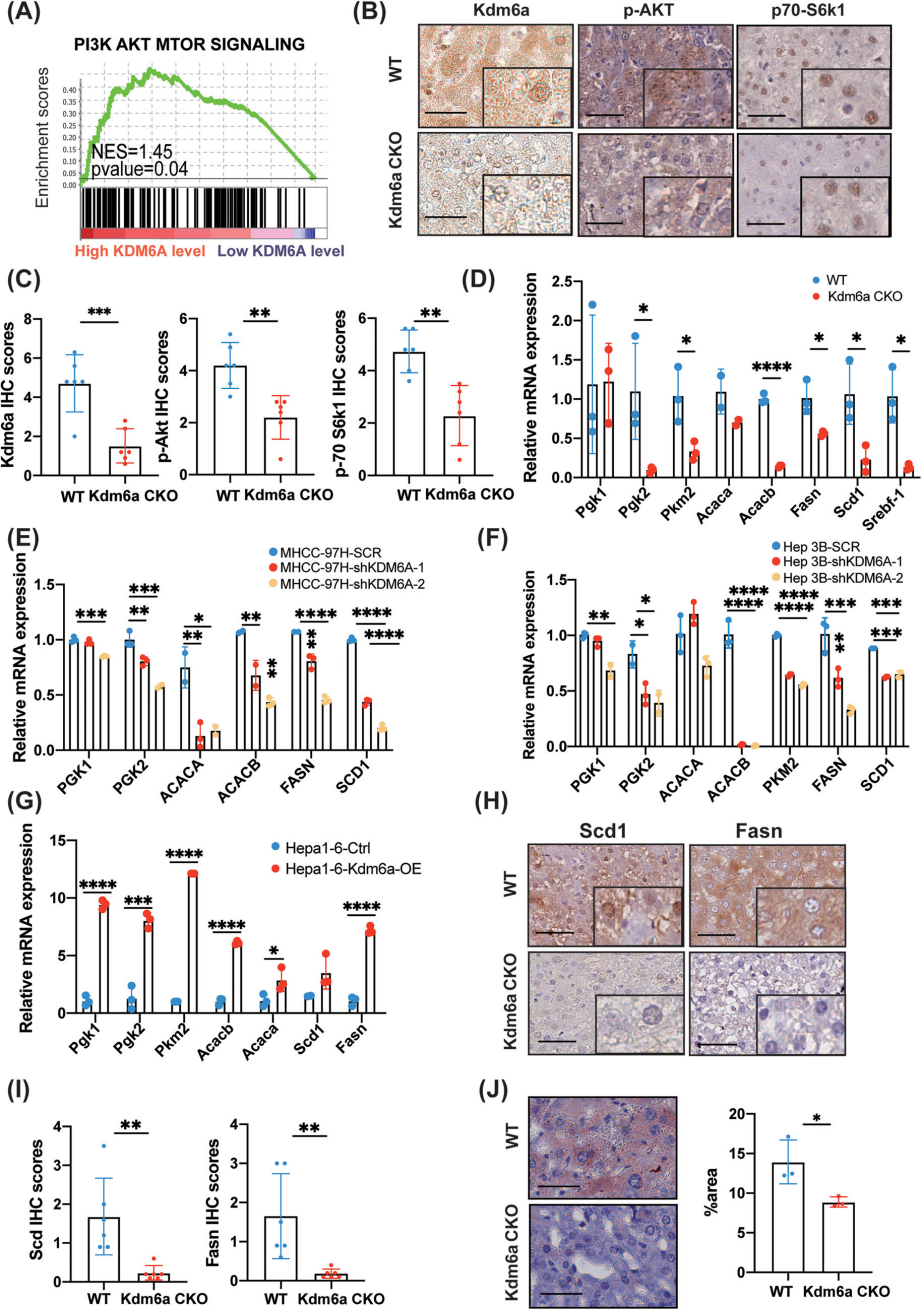
KDM6A plays an important role in HCC lipid metabolism. **(A)** The detailed results of enriched pathways related to PI3K_AKT_MTOR Signaling in TCGA_LIHC (KDM6A high level versus low, n = 185/184). TCGA, The Cancer Genome Atlas; LIHC, liver hepatocellular carcinoma. **(B‐C)** Representative images of IHC and H&E of Kdm6a, p‐AKT and p70‐S6k1staining **(B)** and the relative IHC scores (n = 6) **(C)** in mouse tumour tissues. The IHC scores were assigned according to the percentage of positive staining cells (0−100%) and the staining intensity (0–8). Scale bar, 100 μm. The rectangles within the figure panels signified a magnified image (40×) to better observe the nuclear staining. IHC, Immunohistochemistry; H&E, haematoxylin–eosin staining. **(D)** Relative mRNA level of liver metabolism genes by qPCR in mouse tumour tissues (n = 3). Actin was used as a housekeeping gene for normalization. **(E‐F)** Relative mRNA level of liver metabolism genes by qPCR in MHCC‐97H **(E)** and Hep3B **(F)** cells after KDM6A stable knockdown (n = 3). ACTIN was used as a housekeeping gene for normalization. **(G)** Relative mRNA level of liver metabolism genes by qPCR in Hepa1–6 KDM6A overexpression cell line (n = 3). Actin was used as a housekeeping gene for normalization. **(H‐I)** Representative images of IHC and H&E of Scd1 and Fasn staining **(H)** and the relative IHC scores (n = 6) **(I)** in mouse tumour tissues. Scale bar, 50 μm. The rectangles within the figure panels signified a magnified image (40×). **(J)** Representative images of Oil Red ‘O’ staining and the area ratio (n = 3) in mouse tumour tissues. Scale bar, 50 μm. Data represent mean ± SD. *p* values were calculated using a student *t* test. The significance of multiple group comparisons was analysed by one‐way ANOVA. *p < .05; **p < .01; ***p < .001; ****p < .0001.

FGFRs signalling is a key upstream regulator of the metabolism pathways such as the PI3K–ATK–mTOR pathway in HCC.^23^ We evaluated whether KDM6A could regulate FGFR expression to activate the PI3K–AKT–mTOR signalling, leading to the re‐programming of HCC cell metabolism. Additionally, GSEA showed that the PI3K–AKT–mTOR signalling was activated in the high KDM6A level group of the TCGA_LIHC dataset (Figure 6A), which was consistent with our RNA‐sequencing results (Figure 4A). Furthermore, we also observed a reduction in p‐AKT and p70‐S6k1, in response to decreased PI3K–AKT–mTOR signaling,^23^ in the Kdm6a‐deficient liver tumour tissues based on IHC staining assay (Figure 6B,C).

Considering that the activation of PI3K–AKT–mTOR signalling can promote lipogenesis and glycolysis,^24^ we determined whether KDM6A could also regulate lipid and glucose metabolism re‐programming in HCC. We examined the key genes involved in lipid metabolism (FASN, SCD1, SREBF1, ACACA and ACACB) and glycolysis (PGK1, PGK2 and PKM) to verify whether KDM6A regulated HCC cell lipid and glucose metabolism. As expected, the mRNA levels of these genes were downregulated in the liver tumour tissues of Kdm6a CKO mice (Figure 6D). Similar results were also observed in the KDM6A knock‐down HCC cell lines (Figure 6E,F). In contrast, Kdm6a overexpression cells displayed significantly increased expression of the key genes involving lipid and glucose metabolism compared to control‐transfected cells (Figure 6G). Furthermore, we used IHC and Oil Red ‘O’ staining to evaluate the level of lipid metabolism and we found that Kdm6a‐deficient tumour tissues exhibited a lower staining intensity than the WT tissues (Figure 6H–J). Also, western blot assays further found that knockdown of KDM6A reduced the expression level of p‐S6K1, FASN and SCD1 in MHCC‐97H cells and mouse HCC tissues (Supporting Information Figure S6A,B).

In addition, the levels of KDM6A were positively correlated with those of ACACA, ACACB, SREBF1, FASN and PKM in the HCC tissues from TCGA‐LIHC and CHCC_HBV cohorts (Supporting Information Figure S6C,D). Taken together, our results demonstrate that KDM6A regulates HCC cell lipid and glucose metabolism in HCC cells.

To determine whether KDM6A was dependent on FGFR4 expression to promote HCC progression, we constructed Fgfr4 stable knock‐down cell line while overexpressing Kdm6a in Hepa1–6 (Supporting Information Figure S6E). First, we examined the key genes involved in lipid metabolism (Fasn, Scd1, Srebf1 and Acacb) and glycolysis (Pgk2) to demonstrate whether Kdm6a is dependent on Fgfr4 expression to regulate HCC lipid and glucose metabolism. As expected, we found that the increased lipid and glucose metabolism resulted from Kdm6a overexpression was eliminated by Fgfr4 knockdown (Supporting Information Figure S6F). Besides, Fgfr4 deletion in Hepa1–6 dramatically inhibited HCC cell proliferation ability according to the CCK‐8 assays. However, the effect of Kdm6a‐promoting HCC cell growth could be impaired by Fgfr4 knockdown (Supporting Information Figure S6G). These findings suggested that KDM6A promotes HCC progression mainly via activating FGFR4 expression.

### 3.7 High KDM6A expression enhances lenvatinib sensitivity in response to FGFR inhibition in HCC

**FIGURE 7:**
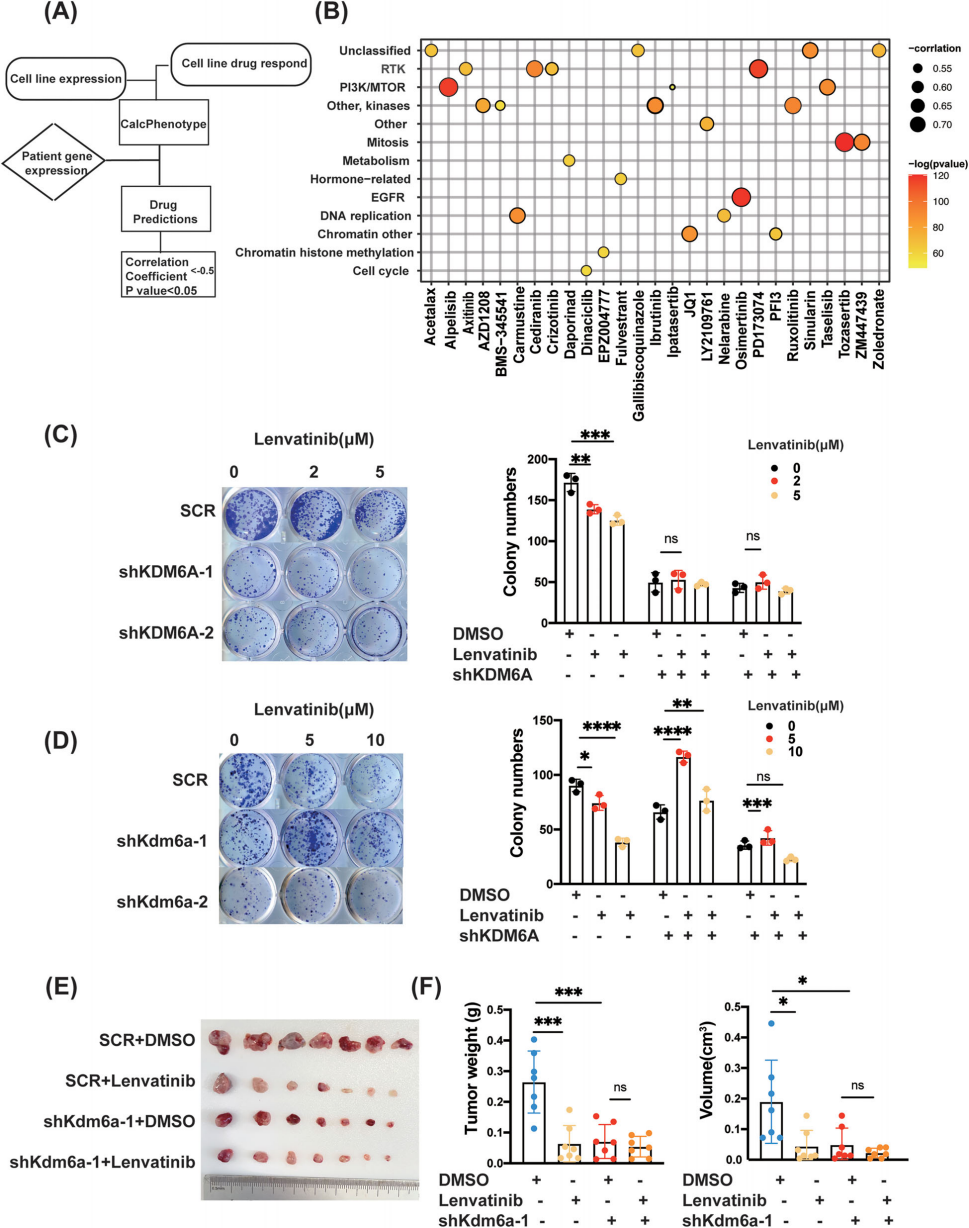
KDM6A knockdown decreases the sensitivity of HCC to lenvatinib treatment. **(A)** Schematic plot about ‘OncoPredict’ R package use. **(B)** Correlation between of KDM6A expression and the drug prediction scores (Correlation Coefficient ← .5, *p* value < .05). Detailed information are in Supporting Information Table S2. **(C‐D)** Colony formation assays of MHCC‐97H **(C)** and Hepa1–6 **(D)** cells in SCR or KDM6A knockdown cells treated with lenvatinib (0–10 μM) or DMSO for 10−14 days (n = 3). **(E)** Photographs of subcutaneous tumours when they were harvested (n = 7 mice per group). **(F)** Subcutaneous Hepa1–6 cells tumour weight and tumour volume in the indicated treatment groups (n = 7 mice per group). Data represent mean ± SD. *p* Values were calculated using a student *t* test. The significance of multiple group comparisons was analysed by one‐way ANOVA. ns, no significance; *p < .05; **p < .01; ***p < .001.

To explore the KDM6A's potential therapeutic implications and possible association with sensitivity to clinical medicines, we first used the ‘OncoPredict’ R package to investigate the associations of KDM6A expression with the sensitivities to the common chemotherapy and molecular targeted drugs and obtained drug prediction scores strongly correlated with the IC50 values of the drugs (Figure 7A‐‐B). We found the most relevant drug targets were RTKs and their downstream PI3K or related signalling genes such as EGFR. Importantly, RTKs inhibitors have been increasingly used in cancer treatment, with lenvatinib being the first‐line systemic treatment for patients with unresectable liver cancer.^3^ This rationale prompted us to explore possible connections between KDM6A and lenvatinib regarding the drug sensitivity. We started this effort by analysing the GEO dataset (GSE186191) in which transcriptomic changes were comprehensively examined and compared between Huh7‐LR (Huh7 lenvatinib resistance) cells and their parental counterparts (Supporting Information Figure S7A). Excitingly, the KDM6A expression was indeed downregulated in the lenvatinib‐resistant HCC cells (Huh7). Moreover, to validate this result, the colony formation assays were conducted and revealed that KDM6A knockdown reduced the sensitivity of HCC cells to lenvatinib treatment in human MHCC‐97H and mouse Hepa1–6 cell lines (Figure 7C,D). To better corroborate these results, we examined the colony formation in Hepa1–6 overexpression cell line and found that the sensitivity of Hepa1–6 cells was enhanced upon the treatment with a low dose of lenvatinib (Supporting Information Figure S7B,C).

To better corroborate these results, we used the LIMORE database^17^ which included the half maximal inhibitory concentration of lenvatinib and KDM6A expression in 31 public cell lines and 50 new cell models established from Chinese liver cancer patients to evaluate the efficacy of lenvatinib in HCC cells with different KDM6A expression. Spearman's correlation analysis revealed a strong negative correlation between KDM6A expression and IC50 of lenvatinib in HCC cells (Supporting Information Figure S7D). The same result was also found in HCC cell lines that says that MHCC‐97H and Hep 3B cells with relative high expression of KDM6A showed lower IC50 of lenvatinib compared to SNU 449 and PLC/PRF/5 with relative low expression of KDM6A (Supporting Information Figure S7E). Besides, we also measured the IC50 of lenvatinib in Kdm6a stable knockdown Hepa1–6 cell lines, and we found that Kdm6a stable knockdown could increase the IC50 of lenvatinib (Supporting Information Figure S7F).

To further investigate the role of KDM6A in lenvatinib sensitivity, we performed an in vivo tumour graft experiment by injecting Kdm6a knockdown (or SCR) Hepa1–6 cells then administered with lenvatinib. Subcutaneous inoculations of 1 × 10^6^ knockdown (or SCR) Hepa1–6 cells were conducted in C57 mice (Supporting Information Figure S7G). Lenvatinib was administered to the mice on alternate days, starting after tumour formation. After 2 weeks, tumour weights and volumes were measured. The scramble control group displayed a strong inhibition of tumour growth, whereas mice bearing the Kdm6a knockdown cell line showed little effect upon lenvatinib treatment, according to the measurements of tumour weight and volume (Figure 7E,F). In conclusion, high KDM6A expression increases the HCC cell sensitivity in response to the RTKs inhibitor, that is, the KDM6A expression level may be a prognostic indicator for the efficacy of lenvatinib in HCC therapy.

## 4 DISCUSSION

HCC is a fatal disease with few limited therapeutic options and has a poor prognosis. Despite the approval of lenvatinib for the treatment of patients with advanced HCC, only a small number of patients can benefit from lenvatinib therapy. Particularly, HCC lacks effective prognostic markers for lenvatinib‐targeted therapies. In this study, we demonstrated that KDM6A was significantly upregulated in HCC and was associated with a poor prognosis. Moreover, we showed that KDM6A promoted liver tumour progression and regulated the re‐programming of liver tumour cell metabolism. Furthermore, we revealed that KDM6A exerted a pro‐oncogene role mainly through activating FGFR4 expression and enhancing the activity of the PI3K‐AKT‐mTOR signalling. Importantly, we indicated that KDM6A enhanced the efficacy of lenvatinib treatment in HCC and proposed that it may be a prognostic biomarker to determine the effectiveness of lenvatinib therapy for HCC.

KDM6A plays various roles as a histone demethylase during cancer development. In support for the present work, several previous studies have reported that KDM6A plays an oncogenic role in tumour progression.^13^, ^25^ Our current findings demonstrated that KDM6A not only promoted the occurrence and development of liver cancer, but also played an important role in targeted therapy for liver cancer. On the other hand, other studies have shown that KDM6A deficiency results in a faster tumour cell growth,^11^, ^26^, ^27^ suggesting that KDM6A is a tumour suppressor. For example, Kdm6a disruption is shown to cause liver tumour formation in conjunction with oncogenesis.^28^ They exploited a mouse HCC model generated by c‐myc overexpression and used Tp53 knockout as a control. However, the authors of this study also found an upregulated mRNA level of KDM6A in HCC tumour tissues, which was consistent with our results.^28^ Additionally, Li et al. used crystal violet and MTT assays to verify that KDM6A inhibited the growth of HCC cell lines (YY‐8103, SNU‐398, Huh7 and LM3) only in vitro.^29^ The discrepancy between our work and the two studies mentioned above is likely due to the use of different animal models and cell lines. These studies also suggest that the function of KDM6A in HCC is very complex and that it may play different roles at different stages of tumour development. Besides, the molecular mechanism of KDM6A‐activated FGFR4 transcription remains to be explored, KDM6A may bind with the transcription factors involved in FGFR4 transcription to perform this function. However, the classical transcription factors involved in FGFR4 transcription are still largely unclear. We used online sites (http://bioinfo.life.hust.edu.cn/hTFtarget#!/target) to predict transcription factors of FGFR4, such as USF1, TAF1 and etc. Whether these transcription factors are involved in the KDM6A‐mediated FGFR4 transcription is one of the subjects of our future research.

Although lenvatinib has gradually become the primary systemic treatment for patients with unresectable liver cancer, the drug resistance to lenvatinib is gradually emerging and studies on drug adaptation in these patients remain highly demanded. In our study, we used the public datasets, colony formation assays and a xenograft liver tumour model to verify that low expression of KDM6A may lead to the resistance to lenvatinib, providing a new strategy for identifying a more adaptive population in the lenvatinib clinical application, which may help to reduce the incidence of resistance in the lenvatinib therapy. In addition, a combination therapy of lenvatinib and anti‐PD‐1 antibody has been recommended as a treatment option for patients with HCC,^30^ suggesting a combination strategy is an alternative way to enhance the lenvatinib sensitivity. Moreover, it is possible that the clinical efficacy achieved by lenvatinib may be attributable to other signalling in addition to RTKs. For example, inhibition of FGFRs by lenvatinib treatment has been reported to activate a feedback of the EGFR/PAK2/ERK5 signalling axis, which can be disrupted by EGFR inhibition.^31^ Therefore, whether KDM6A can also affect other signalling mediating the efficacy of lenvatinib remains to be determined.

It is worth pointing out that although the DEN mouse model employed in the current study has been widely used to induce liver cancer in mice, it has certain limitations. Genetically, the DEN model is considered to be a good representation of HCC associated with a poor prognosis,^32^ but a caveat of this model is that over 80% of DEN‐initiated tumour samples carry an activating hotspot driver mutation in either *Hras* or *Braf*, which human HCC do not harbour during hepatocarcinogenesis.^33^ Moreover, for all mouse models, it needs to be considered that certain signalling pathways in mice may be different from those in humans. Nevertheless, compared to other HCC models, DEN is still a typical, preferred model that appears to recapitulate the occurrence and development of liver cancer for exploring the function of the KDM6A gene.

In summary, this study reports that KDM6A plays an important role in promoting HCC progression. In our animal models, mice with a low KDM6A levels showed a resistance to lenvatinib treatment. As drug response is closely associated with the survival outcome of patients with HCC, our results indicate that KDM6A is an important new molecule affecting the efficacy of lenvatinib in HCC therapy and identify that patients who suffer from HCC with a high KDM6A level are more suitable for lenvatinib therapy.

## CONFLICT OF INTEREST STATEMENT

There is no conflict of interest in this manuscript.

## Supporting information

Suppporting information

Suppporting information

Suppporting information

Suppporting information

Suppporting information

Suppporting information

Suppporting information

Suppporting information

Suppporting information

Suppporting information

## Data Availability

The RNA‐sequencing data can be accessed in NODE (https://www.biosino.org/node) by using the accession code (OEP004319). All data are provided by authors on reasonable request. Supporting Information is available at Clinical and Translational Medicine online.
