# Supplementary figures and images for: KDM6A promotes hepatocellular carcinoma progression and dictates lenvatinib efficacy by upregulating FGFR4 expression
Source: Clin Transl Med. 2023 Oct 17;13(10):e1452. doi: 10.1002/ctm2.1452 (PMC10580016; doi:10.1002/ctm2.1452)

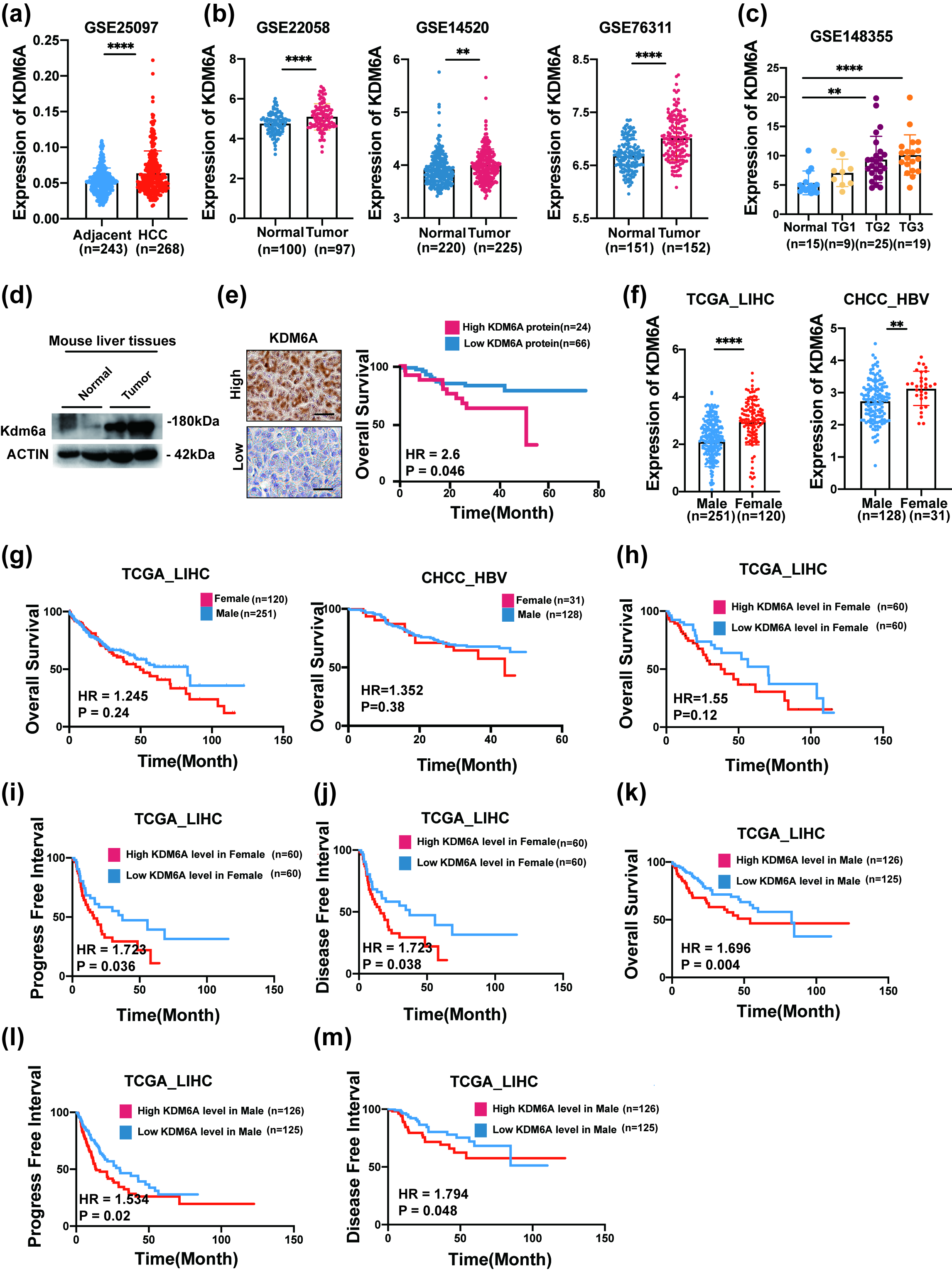

Supplement: Supplementary file 1 — Suppporting information [file CTM2-13-e1452-s003.tif]

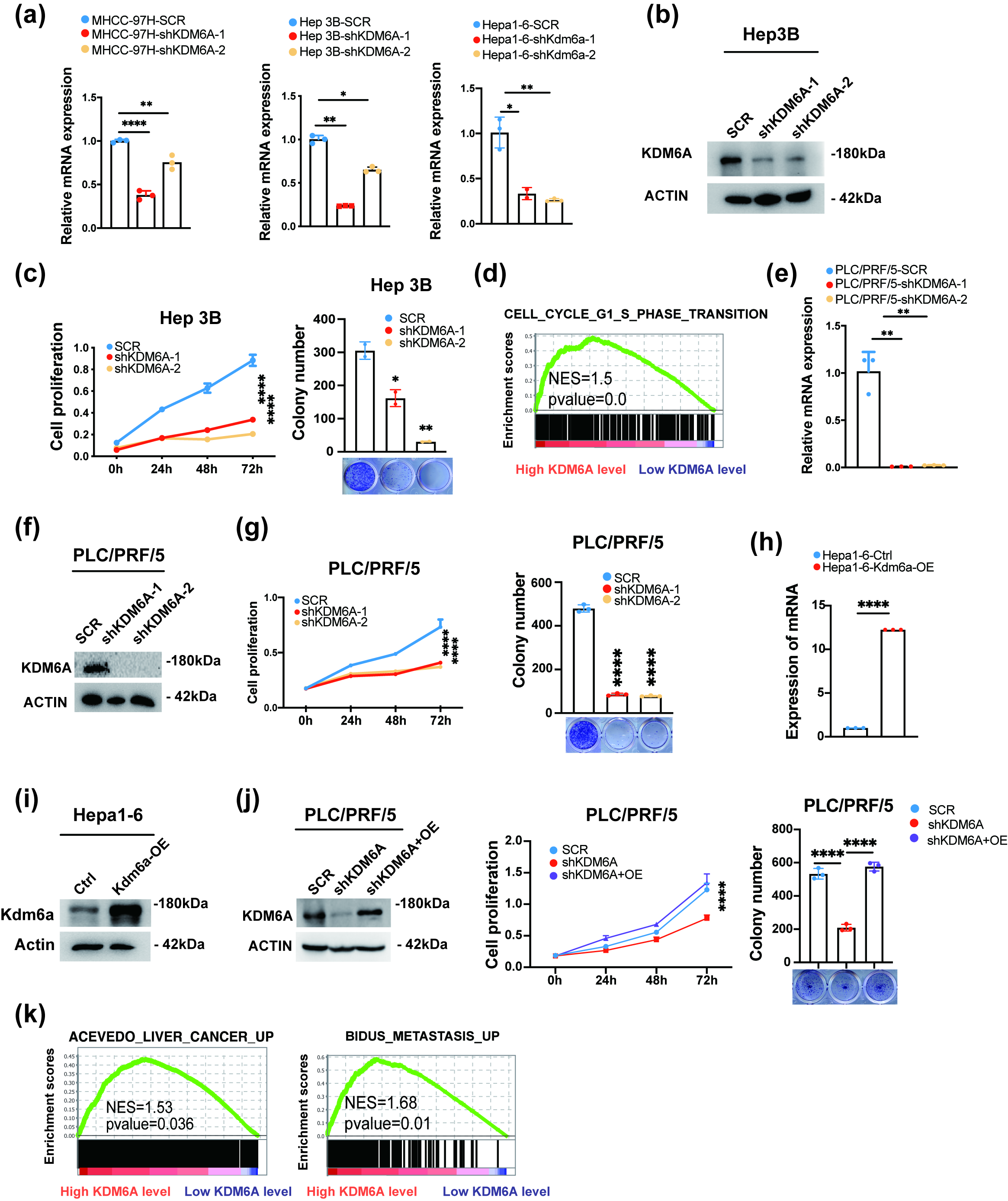

Supplement: Supplementary file 2 — Suppporting information [file CTM2-13-e1452-s001.tif]

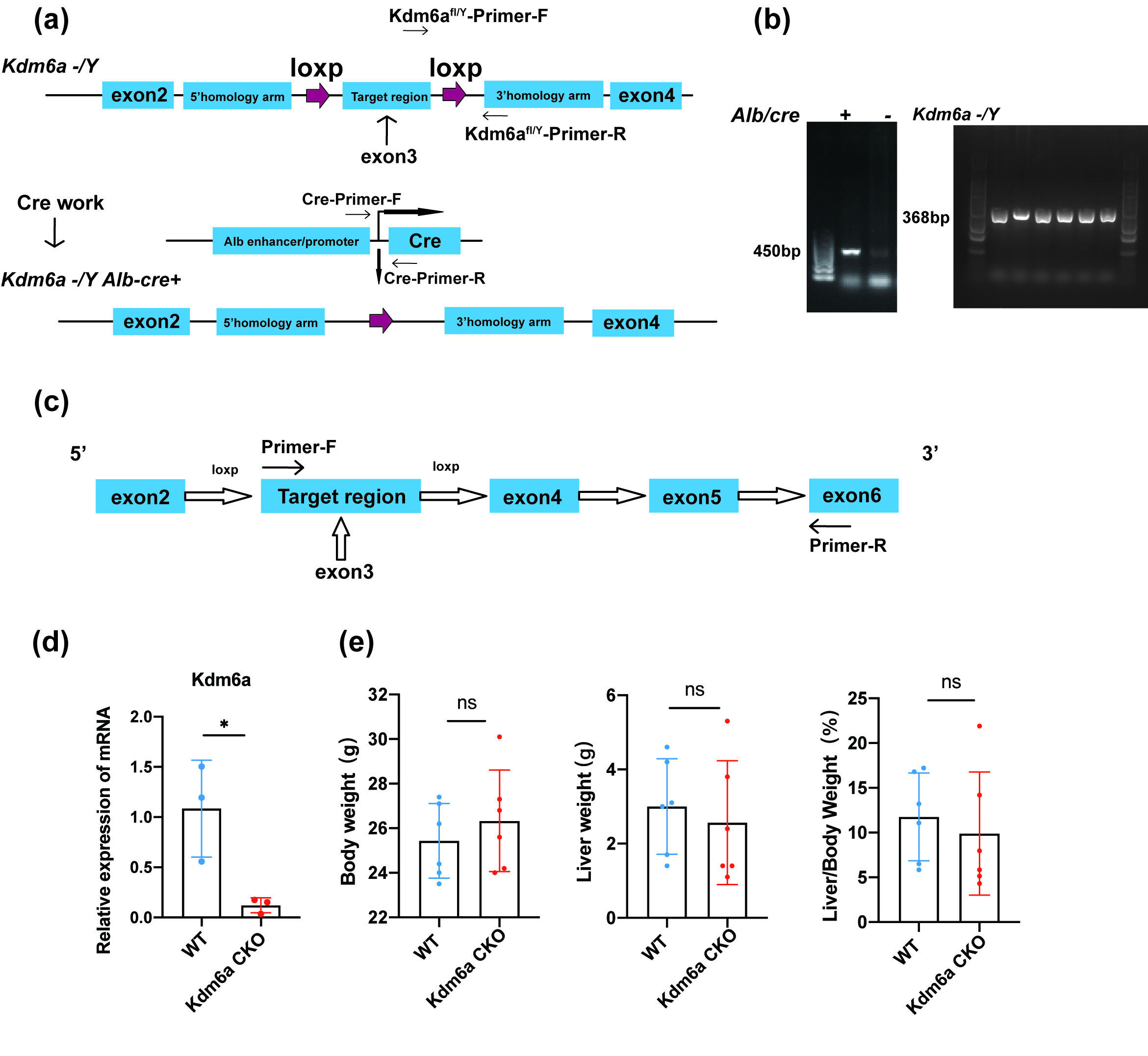

Supplement: Supplementary file 3 — Suppporting information [file CTM2-13-e1452-s006.tif]

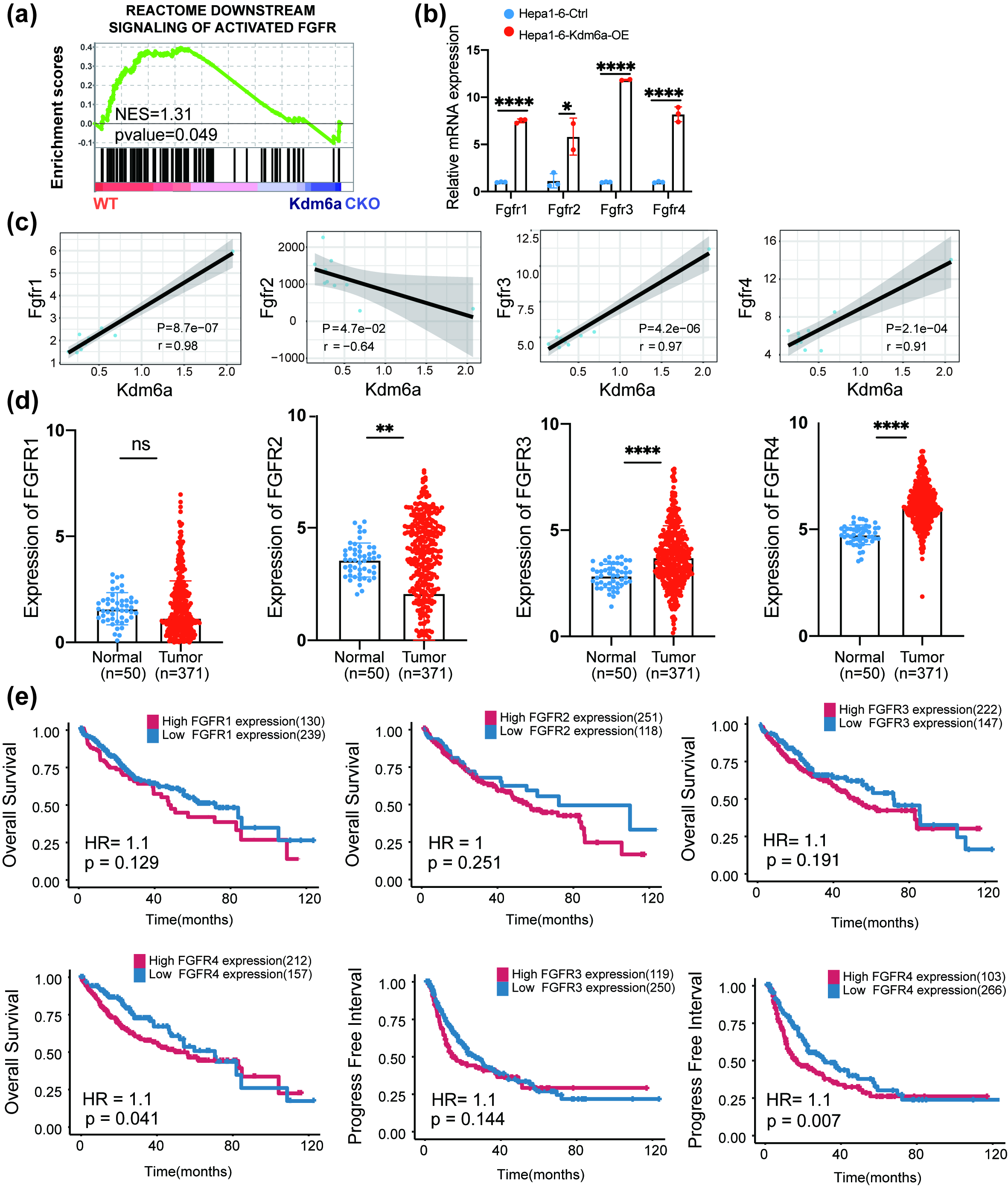

Supplement: Supplementary file 4 — Suppporting information [file CTM2-13-e1452-s005.tif]

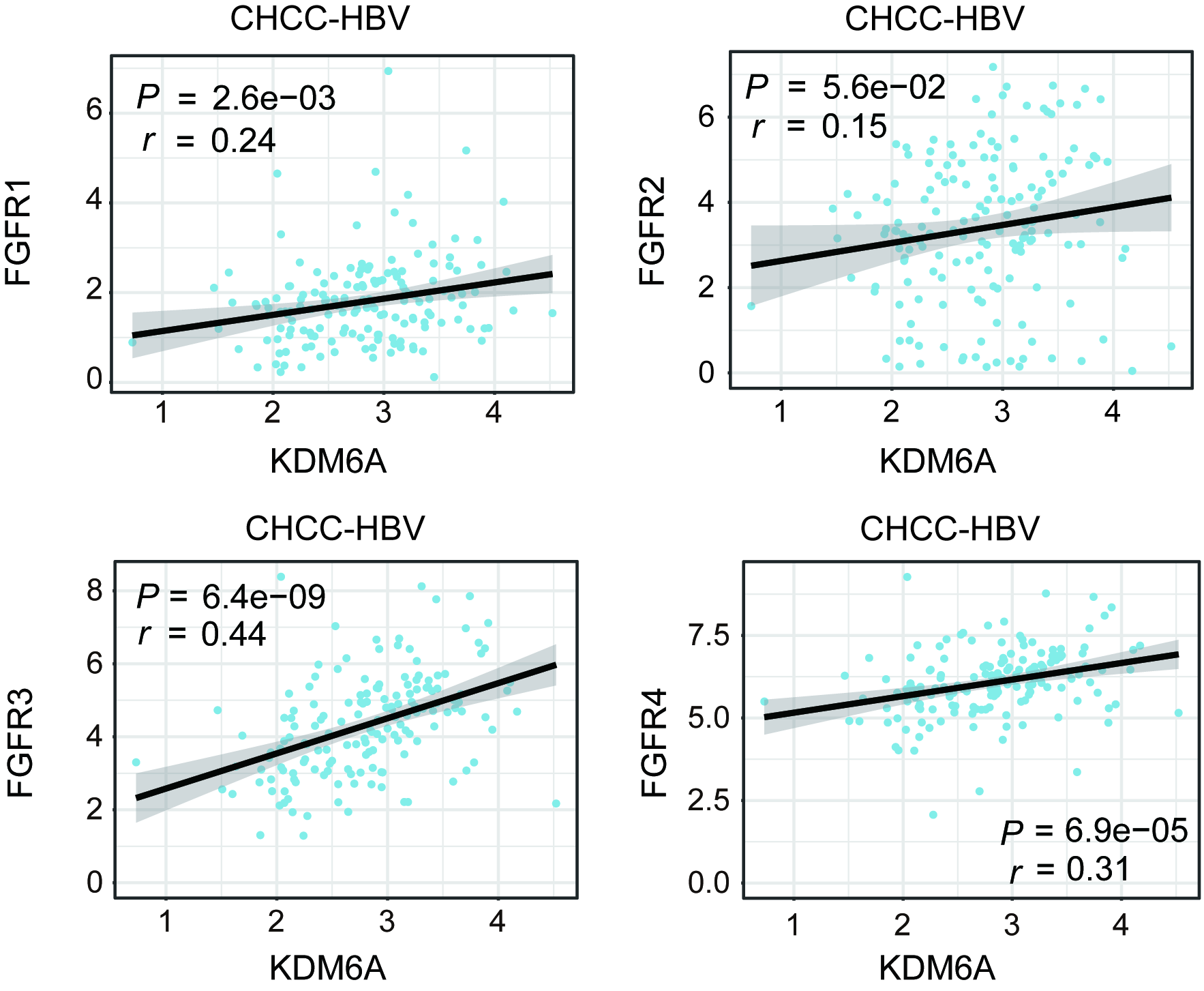

Supplement: Supplementary file 5 — Suppporting information [file CTM2-13-e1452-s007.tif]

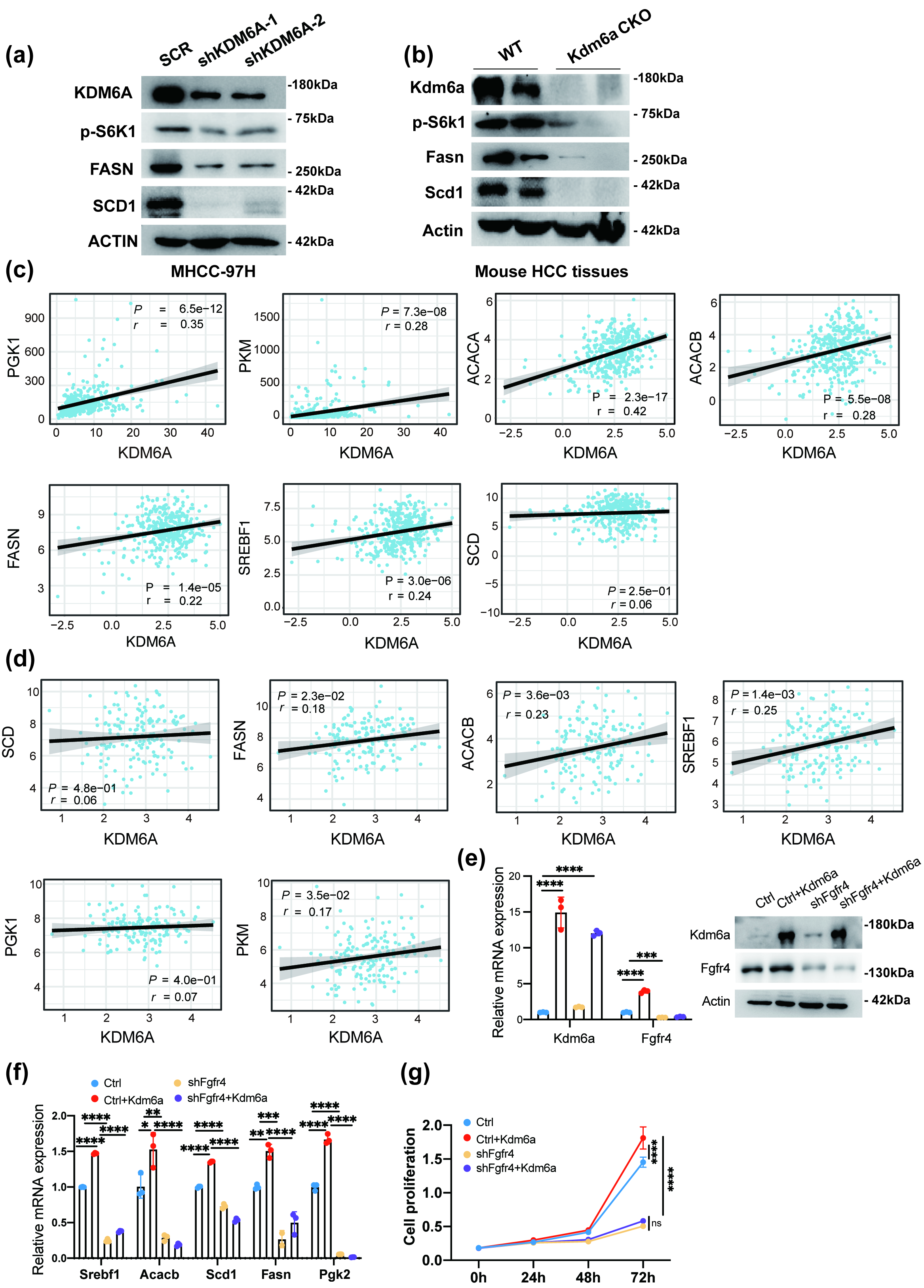

Supplement: Supplementary file 6 — Suppporting information [file CTM2-13-e1452-s008.tif]

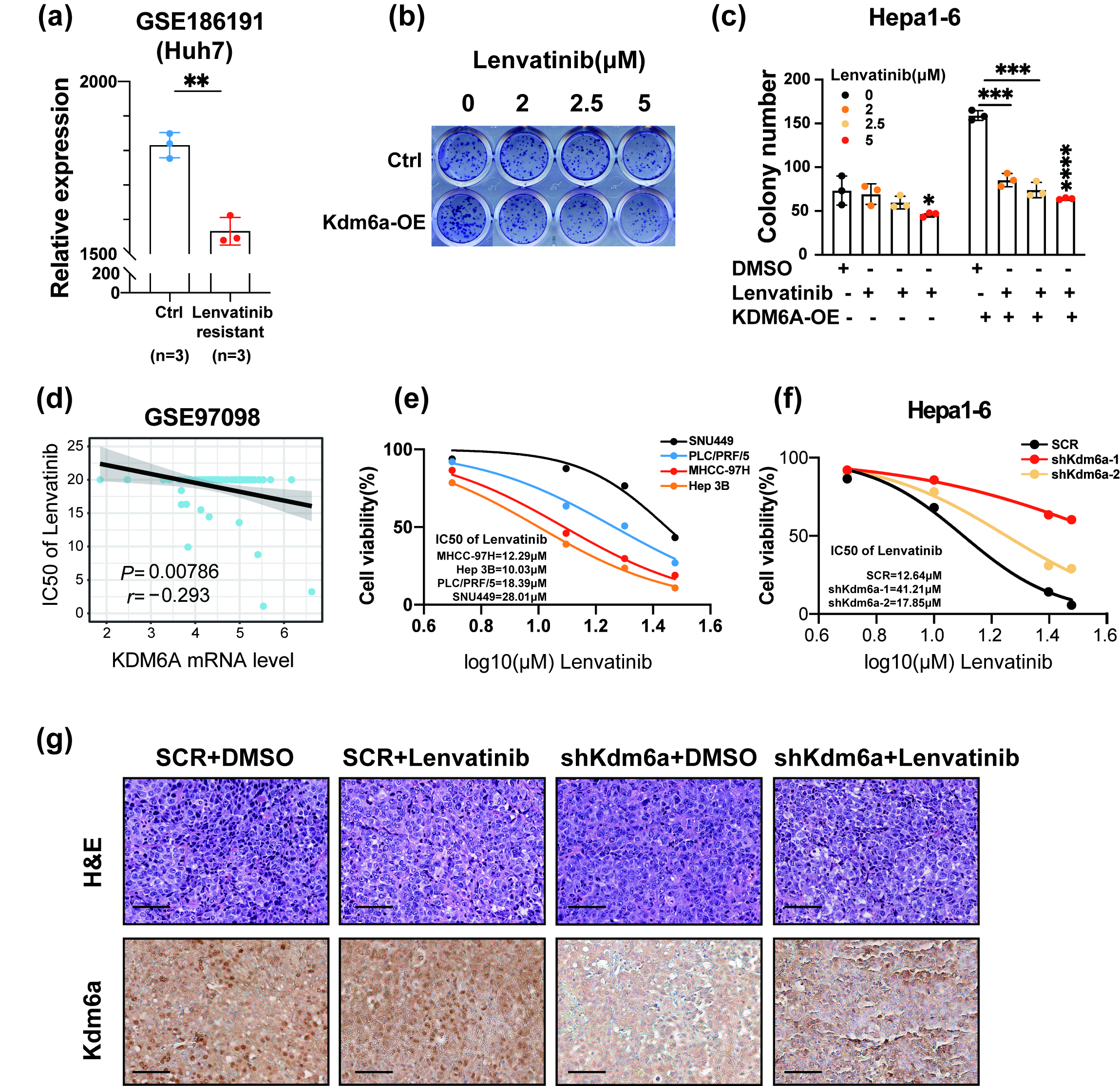

Supplement: Supplementary file 7 — Suppporting information [file CTM2-13-e1452-s004.tif]
