# Supplementary material for: KDM6A promotes hepatocellular carcinoma progression and dictates lenvatinib efficacy by upregulating FGFR4 expression
Source: Clin Transl Med. 2023 Oct 17;13(10):e1452. doi: 10.1002/ctm2.1452 (PMC10580016; doi:10.1002/ctm2.1452)
Supplement: Supplementary file 10 — Suppporting information [file CTM2-13-e1452-s002.docx]

**Supplementary Information**

**Cell culture**

The human liver cancer cell lines, Hep3B, SNU387, SNU398, SNU449, PLC/PRF/5, Hepa1-6 were provided from the American Type Culture Collection (ATCC). MHCC-97H was provided by the Liver Cancer Institute of Zhongshan Hospital.

Hepa1-6 cell line was derived from the BW7756 tumor that arose in a C57/L mouse (https://www.cellosaurus.org/). MHCC-97H was from 39 years old male HCC patient (https://www.cellosaurus.org/). Hep3B was from 8 years old male HCC patient (https://www.atcc.org/). PLC/PRF/5 was from male HCC patient (https://www.atcc.org/). SNU398 was from 42 years male HCC patient (https://www.atcc.org/). SNU449 was from 52 years male HCC patient (https://www.atcc.org/).

All cell lines (Mouse cell lines: Hepa1-6 and Human HCC cell lines: HET-293T, MHCC-97H, Hep 3B, PLC/PRF/5, SNU398) were cultured in DMEM (Gibco) supplemented with 10% fetal bovine serum (FBS) and 1% penicillin/streptomycin.

**RNA extraction and quantitative Real-time PCR (qPCR)**

Total RNA was isolated from cells by FastPure Cell Total RNA Isolation kit (RC112, Vazyme). Complementary DNA (cDNA) was synthesized using a cDNA Reverse Transcription Kit (R333, Vazyme). qPCR was performed using Fast SYBR Green Master Mix (Q711, Vazyme). Fold changes were calculated with the ΔΔCt method using ACTIN as an endogenous control. Gene expression was quantified using primers indicated in Table.S1.

**Cell cycle analysis**

#### Cells were plated in 6 well plates in 50% confluency and incubated for 1-2 days. Then, cells were collected and washed twice with ice-cold PBS. Cells were resuspended in 500µl ice-cold PBS, and 10ml ice-cold ethanol was added dropwise to each sample. Fixed cells were stored in ice for at least two hours and stained 30min by Propidium Iodide (PI) solution with RNaseA at 4°C protected from light. Samples were running on BD AccuriC6, and 30,000-50,000 events were recorded for each sample. FlowJo software was used to analyze the data.

**Cell proliferation and colony formation assay**

In the CCK-8 assay, cells（control and knockdown cells）were seeded in 96 well plates (1000-2000 cells per well) with 200 µl media. After adding 200 µl serum-free DMEM with 10 µl of Cell Counting Kit (CCK8; TARGETMOL) to each well and incubating the plates at 37°C in a humidified 5% CO2 atmosphere for 2-3 hours, a microplate reader was used to measure the absorbance at 450 nm (BioTek Synergy HT).

For colony formation assay, cells (control and knockdown cells）were plated into a 12(or 24) well (1000-2000 cells per well) cell culture plate. For drug sensitivity tests 200-2000 cells per well were plated into a 12 (or 24) well cell culture plate treated with Lenvatinib (0-10µM) (HY-10981; MCE) after being seeded for 24 hours. In 10-14 days, colonies were harvested. Colonies were washed twice with PBS before being fixed for 10-20 minutes at room temperature with 4% Paraformaldehyde (PFA). The fixed colonies were then dyed with 0.005% crystal violet for 20 min. Then colonies were wash twice. The positively stained cells were counted.

**Cell migration**

We used Transwell inserts with an 8µm pore filter (3422, 692 Corning) to perform cell migration assays. The upper chamber of the insert was seeded with 5 x10^4^ cells in 400 µl serum-free medium, and the lower chamber was filled with 600 µl complete medium (DMEM). The cells were collected after 48 hours of incubation. Then, the cells were fixed with 4% paraformaldehyde (PFA) and stained with 0.005% crystal violet. The cells on the bottom surface were examined under a Nikon ECLIPSE 80i microscope at 100 magnifications. We measured the number of migrated cells to evaluate cell migration capacity. 3-5 random fields were photographed for counting purposes. The positively stained cells were counted.

**In vitro drug sensitivity assay**

2 × 10^2^ cells per well were seeded in a 96-well plate. Different concentrations of lenvatinib or DMSO was added to each well in the next day. After HCC cells were treated for 3 days, we added 100 µl serum-free DMEM with 5 µl of Cell Counting Kit (CCK8; TARGETMOL) to each well and incubating the plates at 37°C in a humidified 5% CO2 atmosphere for 2-3 hours. A microplate reader was used to measure the absorbance at 450 nm (BioTek Synergy HT).The half maximal inhibitory concentration value (IC50) for each group was calculated by using Prism 8.0 (GraphPad Software).

**Western blotting**

Total protein was extracted from cells using RIPA Lysis Buffer. Protein was separated via 10% SDS-PAGE and transferred onto nitrocellulose membranes (EMD Millipore). The membranes were blocked with 5% fat-free milk for 1-1.5 h at room temperature. Subsequently, the membranes were incubated with the primary antibodies at 4˚C overnight. The next day, the membranes was blocked by horseradish peroxidase-conjugated secondary antibody for 1 h at room temperature. The immunoreactive protein bands were visualized using chemiluminescent HRP Substrate (Millipore, Cat. No. WBKLS0500) and the ChemiDoc Imaging System (Bio-Rad Laboratories, Inc.). ACTIN (ABclonal, AC028, 1:5000) was used as the loading control. Image Lab software (version 6.0.1) was used for analysis of western blotting.

**Antibody**

The antibodies we used in the article are as follows: anti-KDM6A(Cell Signaling Technology, #33510, 1:1000, Western blot); ACTIN (ABclonal, AC028, 1:5000, Western blot); anti-rabbit IgG (ABclonal, AS014, 1:5000, Western blot); anti-mouse IgG (ABclonal, AS003, 1:5000, Western blot,1:200,IHC); Kdm6a (ABclonal, A8159, 1:200, IHC); PCNA (Proteintech,10205-2-AP,1:1000, IHC); FGFR3 (Santa Cruz,SC-13121,1:200, IHC); FGFR4 (Santa Cruz,SC136988,1:200, IHC,1:500,Western blot ); H3K27me3(ABclonal,A2363,1:100). Kdm6a (ABclonal, A8159,1:200, IHC); p70-S6k1(ABclonal, AP0502,1:100, IHC); FASN (ABclonal, A6273,1:200, IHC) SCD1(ABclonal, A16429,1:200, IHC); p-AKT(Proteintech,66444-1-1g,1:200, IHC);

**List of abbreviations**

CCK-8, Cell Counting Kit-8; OS, overall survival, PFI, progress-free interval; GEO, Gene expression omnibus; GSEA, Gene Set Enrichment Analysis; HCC, hepatocellular carcinoma; IC50, half maximal inhibitory concentration; IHC, immunohistochemistry; LIHC, Liver hepatocellular carcinoma; TCGA, The Cancer Genome Atlas; CHCC_HBV, datasets from Integrated Proteogenomic Characterization of HBV-Related Hepatocellular Carcinoma; RTK, receptor tyrosine kinase; IC50, The half maximal inhibitory concentration.
